# A TMPRSS6-inhibiting mAb improves disease in a **β**-thalassemia mouse model and reduces iron in healthy humans

**DOI:** 10.1172/jci.insight.191813

**Published:** 2025-06-23

**Authors:** Heinrich E. Lob, Nikhil Singh, Kusha Mohammadi, Larisa Ivanova, Beth Crowell, Hyon J. Kim, Leah Kravets, Nanditha M. Das, Yonaton Ray, Jee Hae Kim, Sylvie Rottey, Emily Labriola-Tompkins, Hazem E. Hassan, Lorna Farrelly, Harvey F. Chin, Marilena Preda, Leigh Spencer Noakes, Kei Saotome, Matthew Franklin, Marc W. Retter, Elif Karayusuf, John J. Flanagan, William Olson, Kalyan C. Nannuru, Vincent Idone, Michael E. Burczynski, Olivier A. Harari, Lorah Perlee, Griet Van Lancker, Andrew J. Murphy, Aris N. Economides, Sarah J. Hatsell

**Affiliations:** 1 Regeneron Pharmaceuticals, Tarrytown, New York, USA.; 2 Drug Research Unit Ghent, Ghent University Hospital, Ghent, Belgium.

**Keywords:** Genetics, Hematology, Immunology, Genetic diseases, Immunotherapy

## Abstract

β-Thalassemia is a genetic disorder arising from mutations in the β-globin gene, leading to ineffective erythropoiesis and iron overload. Ineffective erythropoiesis, a hallmark of β-thalassemia, is an important driver of iron overload, which contributes to liver fibrosis, diabetes, and cardiac disease. Iron homeostasis is regulated by the hormone hepcidin; BMP6/hemojuvelin–mediated (BMP6/HJV-mediated) signaling induces hepatic hepcidin expression via SMAD1/5, with transmembrane serine protease 6 (TMPRSS6) being a negative regulator of HJV. Individuals with loss-of-function mutations in the *TMPRSS6* gene show increased circulating hepcidin and iron-refractory iron-deficiency anemia, suggesting that blocking TMPRSS6 may be a viable strategy to elevate hepcidin levels in β-thalassemia. We generated a human mAb (REGN7999) that inhibits TMPRSS6. In an Hbb^th3/+^ mouse model of β-thalassemia, REGN7999 treatment led to significant reductions in liver iron, reduced ineffective erythropoiesis, and showed improvements in RBC health, running distance during forced exercise, and bone density. In a phase I, doubleblind, randomized, placebo-controlled study in healthy human volunteers (NCT05481333), REGN7999 increased serum hepcidin and reduced serum iron with an acceptable tolerability profile. Our results suggest that, by both reducing iron and improving RBC function, inhibition of TMPRSS6 by REGN7999 may offer a therapy for iron overload and impaired erythropoiesis in β-thalassemia.

## Abstract 2

Antibody blockade of TMPRSS6 significantly reduces liver iron and improves red blood cell health, resulting in increased physical fitness in a beta-thalassemia mouse model.

## Introduction

β-Thalassemia is an inherited anemia caused by mutations in the β-globin gene, which leads to reduced production of β-globin (1). Under physiological conditions, hemoglobin consists of a tetramer of 2 α- and 2 β-globins; the decreased synthesis of β-globin in β-thalassemia results in unpaired α-globin contributing to oxidative stress and RBC senescence (2–4).

Increased iron demand during erythropoiesis leads to suppression of the main iron regulatory hormone hepcidin (gene: *HAMP* in primates; *Hamp1* in mice) (5–8). Hepcidin production is positively regulated by BMP6/hemojuvelin (HJV) signaling and negatively regulated by transmembrane protease, serine 6 (TMPRSS6). During increased erythropoietic demand, the hormone erythroferrone is secreted by erythropoietic precursors; it suppresses hepatic BMP6 signaling and hepcidin secretion (9, 10). Hepcidin engages ferroportin (the only cellular iron exporter) and causes its internalization and degradation (11). High levels of hepcidin reduce membrane ferroportin, trapping iron within macrophages and enterocytes and potentially causing anemia. In contrast, inappropriately low levels of hepcidin result in increased dietary iron uptake and transferrin saturation. Chronic suppression of hepcidin, as observed in β-thalassemia, leads to iron overload (12). In addition to the hyperabsorption of iron, blood transfusions, which are lifesaving for many β-thalassemia patients, further contribute to iron overload (13).

Iron is normally transported by transferrin as transferrin-bound iron, but during states of iron overload, it is also present as non-transferrin-bound iron. Iron enters the cells in its ferrous form and is normally stored by ferritin or in the labile iron pool (14); ferritin stores iron in its ferric form to prevent oxidative reactions and cellular damage (15). Chronic uptake of non-transferrin-bound iron exhausts the ferritin stores, resulting in more iron being stored in the labile iron pool. In addition, iron export via ferroportin and transferrin is limited, since transferrin saturation is already high and, thus, more iron accumulates as free iron in the labile iron pool, which is highly reactive and contributes to oxidative stress. Consequences of elevated oxidative stress can be cardiac toxicity, liver fibrosis and cirrhosis, metabolic dysfunction, hypogonadism, and hypothyroidism (16). Removing excessive iron is therefore important in β-thalassemia to mitigate the comorbidities associated with iron overload.

The current standard therapy to reduce iron overload in β-thalassemia is iron chelation, which has improved the life span and health of patients (17). However, iron chelators have significant toxicities, leading to patient dissatisfaction and nonadherence, potentially limiting their efficacy (18, 19). Erythropoietic stimulating agents such as luspatercept [ACVR2B(L79D)-Fc], can significantly reduce transfusion burden but have not been shown to cause clinically significant liver iron reduction (20, 21). Gene therapies are showing promise for the treatment of hemoglobinopathies including β-thalassemia; however, ineffective erythropoiesis is not restored to baseline in many patients, suggesting a potential need for chronic iron-reducing therapy (22).

Targeting the BMP6/HJV/hepcidin axis to increase hepcidin is an attractive way to reduce iron uptake, and this has received wide attention in recent years. Minihepcidins, synthetic human hepcidin, and oral ferroportin inhibitors show efficacy in reducing iron loading in mouse models and in humans (clinicaltrials.gov; NCT04057040) (23, 24). An alternative strategy is to blunt TMPRSS6 expression or function to increase BMP6/HJV signaling activity. Human genetics clearly support the development of modulators of this pathway, as patients with loss-of-function mutations in *TMPRSS6* show high hepcidin levels and have iron-refractory iron-deficient anemia (25). Additionally, non-transfusion-dependent β-thalassemia patients with loss-of-function mutations in *TMPRSS6* show reduced liver iron (26). Antisense oligonucleotides (ASO) targeting *TMPRSS6* were successfully tested in phase I (27) and recently underwent phase II clinical testing (clinicaltrials.gov; NCT04059406). We therefore explored whether inhibition of TMPRSS6 by using a mAb that binds to the serine-protease domain and prevents cleaving of HJV would be an effective way of reducing iron overloading. Our strategy improves both iron loading and RBC health in β-thalassemia. This dual effect is unlike current treatments, which either reduce iron loading or improve RBC health but not both.

## Results

### REGN7999 binds to several different forms of TMPRSS6 and blunts its activity in vitro.

To inhibit TMPRSS6, we generated a human mAb, REGN7999. We assessed the ability of REGN7999 to block TMPRSS6-mediated cleavage and release of HJV from the cell membrane. REGN7999 blunted soluble HJV levels in the supernatant of cells overexpressing HJV and TMPRSS6, confirming that this mAb blocks the function of TMPRSS6 (Supplemental Figure 1, A and B; supplemental material available online with this article; https://doi.org/10.1172/jci.insight.191813DS1). We confirmed these findings by measuring retention of cell surface HJV by flow cytometry. Isotype control-treated HEK293.hHJV.hTMPRSS6 or HEK293.hHJV.mTMPRSS6 cells lacked HJV surface staining (Supplemental Figure 1, C and D). Cells treated with aprotinin (a protease inhibitor used as a positive control) or REGN7999 had high staining for HJV, showing that aprotinin and REGN7999 prevent cleavage of HJV.

REGN7999 binds murine, cynomolgus monkey, and all tested human TMPRSS6 reference and commonly occurring TMPRSS6 variants (V736A, K253E, and P555S) (26, 28) with similar EC_50_ (Supplemental Figure 2), and it inhibits TMPRSS6 activity by > 90%. The IC_50_ ranged from 4.10 to 6.92 nM dependent on species and variant (Supplemental Figure 3), while the isotype control Ab showed little to no inhibition of TMPRSS6 cleavage activity.

To investigate the mechanism of inhibition, we used cryogenic electron microscopy (Cryo-EM) to determine a structure of TMPRSS6 in complex with the Fab fragment of REGN7999. We found that REGN7999 does not appear to directly occlude the catalytic triad and likely inhibits TMPRSS6 activity allosterically by binding the LDLRA2-domain and part of the catalytic domain (Supplemental Figure 4).

### REGN7999 lowered serum iron and increased serum hepcidin in preclinical studies.

**Figure 1:**
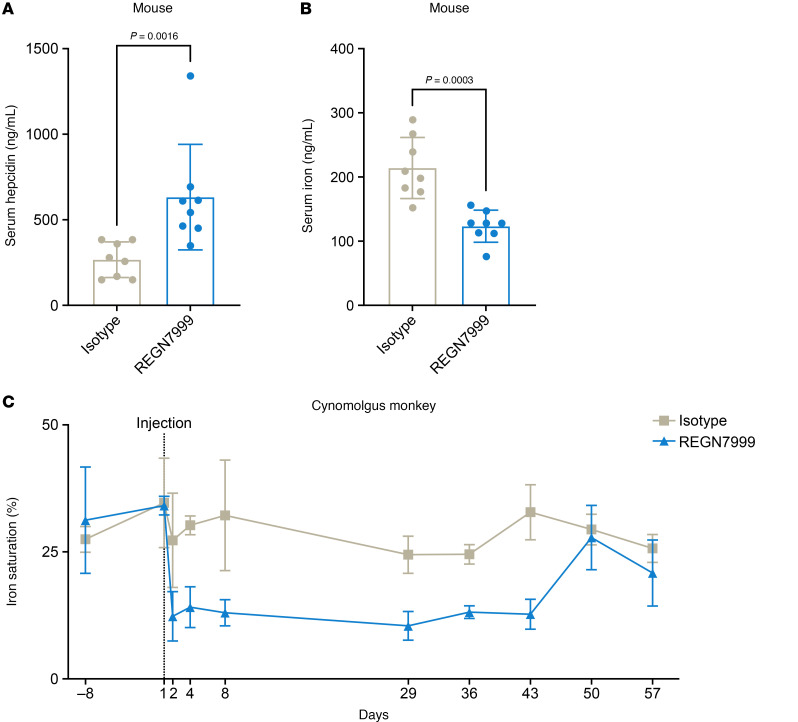
TMPRSS6 inhibition modulates iron levels in mice and nonhuman primates. (**A** and **B**) Serum hepcidin and serum iron in C57BL/6 3 days after injection with REGN7999 or isotype control. (**C**) Iron saturation in primates after 1 bolus injection of REGN7999 or isotype control. Significance at *P* < 0.05 by Student’s *t* test (2-tailed).

We tested whether REGN7999 reduces serum iron and increases hepcidin levels in rodents and nonhuman primates (NHPs). Three days after a 5 mg/kg injection of REGN7999, WT mice showed significant increases in serum hepcidin (Figure 1A) and decreases in serum iron (Figure 1B), versus isotype control, demonstrating that REGN7999 is efficacious in vivo. In cynomolgus monkeys, REGN7999 administration led to a significant drop of transferrin saturation and serum iron in the first 24 hours (Figure 1C and Supplemental Figure 5A). Serum hepcidin concentrations in cynomolgus monkeys were highly variable but similar throughout the study across subjects. In primates, circadian regulation of hepcidin expression and diurnal alterations in iron uptake contribute to hepcidin variations (29–31), which were also observed in the NHPs used in this study (Supplemental Figure 5B).

### REGN7999 increases hepcidin in Hbb^th3/+^ mice.

**Figure 2:**
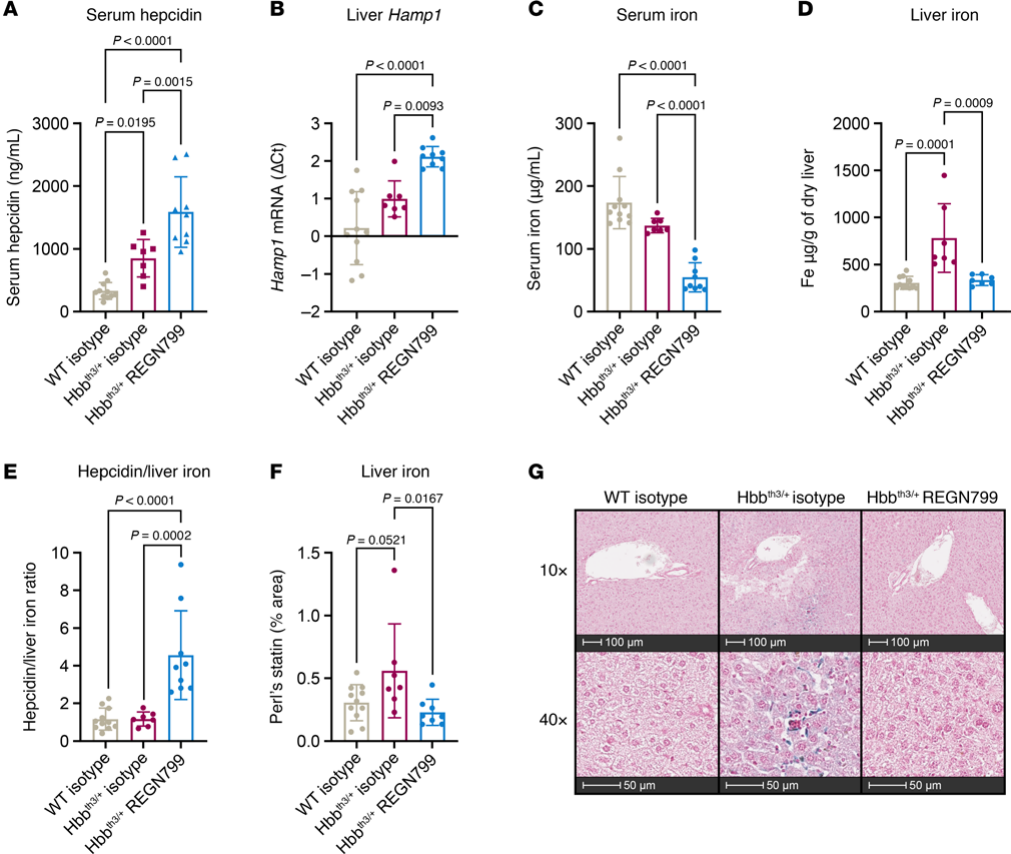
REGN7999 normalizes liver iron loading in Hbb^th3/+^ mice after 8 weeks. (**A**–**C**) Serum hepcidin, liver *Hamp1* (presented as -DCt), and serum iron of Hbb^th3/+^ or WT mice after 12 weeks of treatment with REGN7999 or isotype control. (**D**–**F**) Liver iron content and the ratio of hepcidin/liver iron of the same groups of mice measured using a modified version of Bothwell and Torrance (49) (**D** and **E**) or Perl’s stain (**F**). (**G**) Representative images from these livers at 10× and 40× magnification. Scale bars: 100 μm (top row), 50 μm (bottom row). Blue stain indicates iron accumulation. *P* < 0.05 was significantly different. Significance was determined by 1-way ANOVA with Tukey’s multiple-comparison test.

Given the reductions in serum iron and increases in hepcidin levels in nonthalassemic rodents and NHPs, we tested whether REGN7999 can normalize iron levels (or iron homeostasis) in a mouse model of β-thalassemia. We used Hbb^th3/+^ mice, a mouse model of β-thalassemia intermedia. These animals display increased reticulocyte count, low RBC count, and low hemoglobin, and they show a significant liver iron overload (32). We injected REGN7999 into Hbb^th3/+^ mice for up to 12 weeks (10 mg/kg weekly; in the first week the mice received 2 injections). Body and liver weights remained similar between the groups (Supplemental Figure 6, A and B). As anticipated, REGN7999 significantly elevated serum hepcidin and hepatic *HAMP1* expression in Hbb^th3/+^ mice compared with isotype control (Figure 2, A and B), while serum iron was significantly reduced (Figure 2C). Alanine and aspartate aminotransferases were similar between WT and Hbb^th3/+^ mice and REGN7999 treatment did not increase markers of liver damage (Supplemental Figure 7, A and B).

### REGN7999 reduces liver iron but not splenic iron in Hbb^th3/+^ mice.

Liver and splenic iron overload are hallmarks of β-thalassemia and are replicated in Hbb^th3/+^ mice. Treatment with REGN7999 reduced hepatic iron (Figure 2, D, F, and G). The hepcidin/liver iron ratio was significantly increased after REGN7999 treatment (Figure 2E). Treatment with REGN7999 did not significantly reduce splenic iron concentration; however, as the spleen is significantly reduced in size, the total amount of splenic iron is lowered (Supplemental Figure 8, A–D). This suggests that iron is retained within the spleen, presumably in spleen-resident macrophages (see below).

Furthermore, we tested whether REGN7999 blocks iron uptake, by measuring duodenal iron. We found that duodenal iron was unchanged, suggesting that, despite high hepcidin and likely low ferroportin expression, some iron still enters the body (Supplemental Figure 8, E and F).

### REGN7999 normalizes erythropoiesis in Hbb^th3/+^ mice.

**Figure 3:**
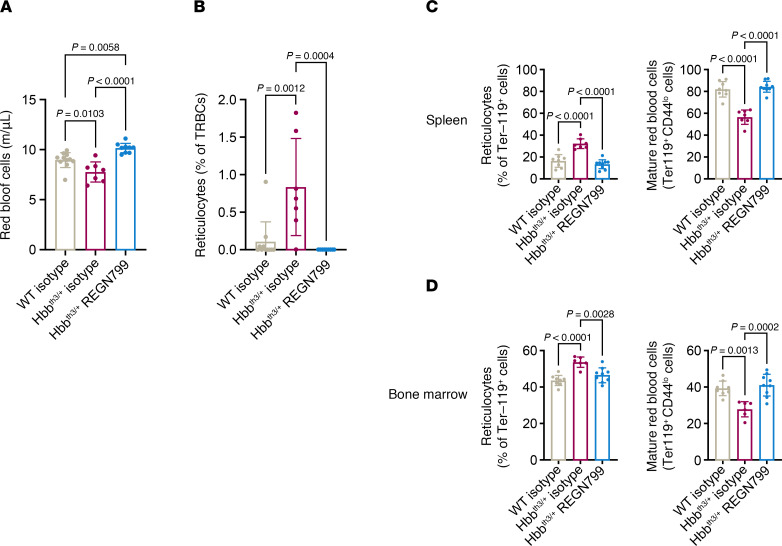
REGN7999 treatment improves erythropoiesis in Hbb^th3/+^ mice. (**A** and **B**) Complete blood count (CBC) was performed to monitor RBCs (**A**) and reticulocytes (**B**) in Hbb^th3/+^ mice and control littermates after 8 weeks of treatment. (**C** and **D**) Flow cytometry in spleen and bone marrow to measure reticulocytes and mature RBCs was performed to confirm the CBC findings. Significance (*P* < 0.05) was determined by 1-way ANOVA with Tukey’s multiple-comparison test.

Compared with WT littermates, Hbb^th3/+^ mice have reduced RBC numbers, smaller mean RBC corpuscular volume (MCV), and lower hematocrit and hemoglobin, while their reticulocyte number is significantly increased. Whereas treatment with isotype-control had no effect on the RBC parameters, REGN7999 treatment normalized RBC (Figure 3A) and reticulocyte numbers (Figure 3B), indicating improved erythropoiesis (Table 1).

The spleen is a site of secondary erythropoiesis in β-thalassemia, with splenomegaly being a marker of extramedullary hematopoiesis. Notably, splenomegaly is considered a clinically relevant phenotype (16) and is frequently surgically addressed with splenectomy. Mirroring patients with β-thalassemia, the spleens of Hbb^th3/+^ mice were approximately 4-fold heavier than WT spleens. Treatment with REGN7999 reduced spleen weights to WT levels (Supplemental Figure 8C).

In order to understand whether this effect is due to normalized erythropoiesis, we used flow cytometry to assess erythroblast maturation in the spleen and at the primary site of erythropoiesis, bone marrow. Spleens and bone marrow of Hbb^th3/+^ mice had increased numbers of reticulocytes compared with WT littermates, whereas Hbb^th3/+^ mice treated with REGN7999 displayed normalized reticulocyte numbers (Figure 3C). Hbb^th3/+^ mice also display a significant reduction of mature RBCs compared with WT mice, mirroring what is observed in human β-thalassemia. Treatment with REGN7999 normalizes the number of mature RBCs in spleens and bone marrow in Hbb^th3/+^mice (Figure 3D). These results are consistent with a mechanism whereby inhibition of TMPRSS6 normalizes erythropoiesis in thalassemic mice.

### REGN7999 extends RBC lifespan and improves RBC health in Hbb^th3/+^ mice.

**Figure 4:**
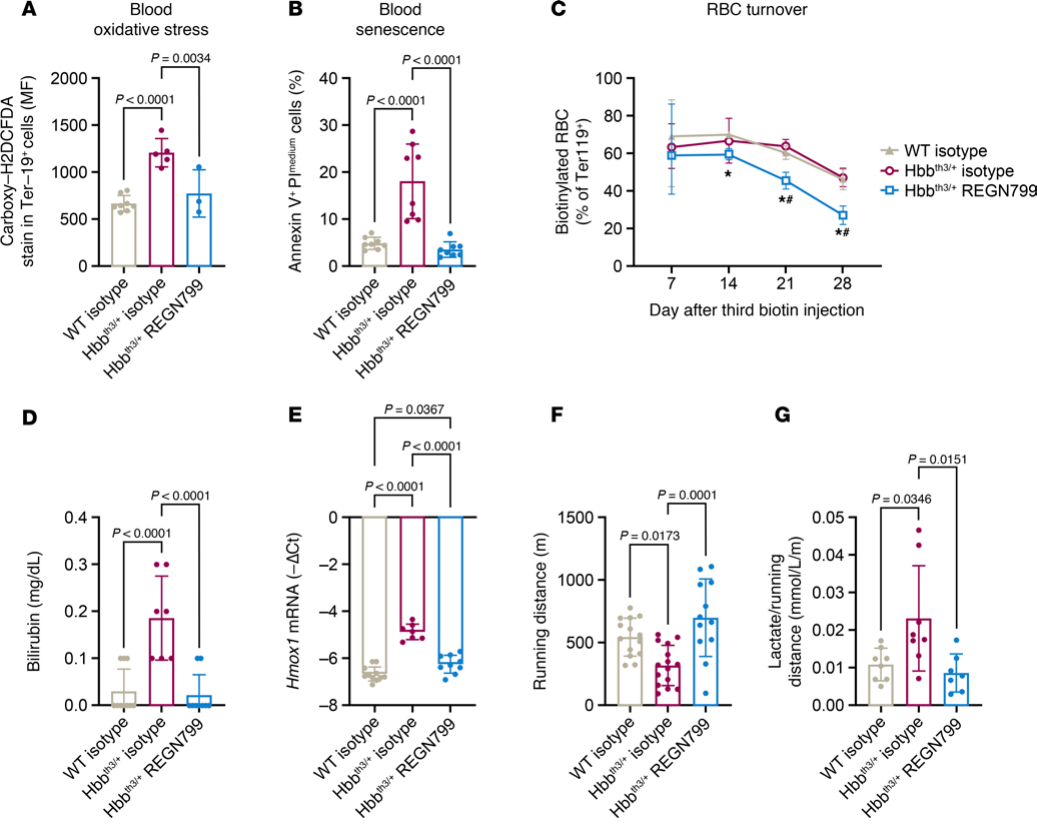
REGN7999 treatment results in healthier RBCs. In all studies, we compared Hbb^th3/+^ mice with WT littermates after or during treatment with REGN7999 or isotype control. (**A**) Carboxy-H2DCFDA levels in whole blood of Hbb^th3/+^ or littermate WT controls to measure oxidative stress. (**B**) Annexin V staining in whole blood to discover RBC senescence. (**C**) Biotinylated RBCs measured over 28 days (week 8–12 of the study) to determine RBC turnover. (**D** and **E**) Serum bilirubin levels and liver Hmox1 mRNA were measured to determine RBC destruction. (**F**) The distance that the test mice ran during a forced exhaustion run. (**G**) The ratios of blood lactate levels measured directly at exhaustion and running distance to receive lactate produced per meter running. Carboxy-H2DCFDA, 6-carboxy-2’,7’-dichlorodihydrofluorescein diacetate. Significance was given at *P* < 0.05 and determined by 1-way ANOVA (**A**, **B**, and **D**–**G**) or 2-way ANOVA (**C**) with Tukey’s multiple-comparison test.

The increase in RBCs and reduction of reticulocytes suggests that mature RBCs live longer in response to TMPRSS6 inhibition. Previous studies suggest that iron restriction blunts oxidative stress and slows RBC senescence (33, 34). We used carboxy-H2DCFDA to measure oxidative stress in blood, spleen, and bone marrow, and we found that oxidative stress was elevated in the blood (Figure 4A) and spleens (Supplemental Figure 9A) but not in the bone marrow of Hbb^th3/+^ mice (Supplemental Figure 9B). Treatment with REGN7999 blunted the increase in oxidative stress in blood (Figure 4A) and spleens (Supplemental Figure 9A).

Increased oxidative stress causes damage to the cell membrane, which in turn may accelerate RBC senescence. Damage to the cell membrane can be measured via annexin V binding. Hbb^th3/+^ RBCs display significantly increased annexin V binding compared with RBCs from WT mice, suggesting elevated RBC senescence in blood (Figure 4B), spleen, and bone marrow (Supplemental Figure 9, C and D). REGN7999 treatment blunted annexin V staining on the extracellular side of Hbb^th3/+^ RBCs in blood and spleens (Figure 4B and Supplemental Figure 9C). Similar results were seen in bone marrow, but the level of correction did not reach statistical significance (Supplemental Figure 9D).

We next assessed whether REGN7999 treatment would extend the RBC life span. For this, we injected NHS-PEG-NHS-biotin into mice, labeling RBCs and allowing an estimate of cell turnover. In line with a prior study (23), Hbb^th3/+^ mice showed a significant reduction of biotinylated RBCs after 14 days; at the end of the experiment (28 days), these animals only had approximately 30% of biotinylated RBCs. In WT animals, approximately 50% of RBCs were still labeled with biotin. Hbb^th3/+^ mice treated with REGN7999 showed virtually the same levels of biotinylated RBCs as WT mice, indicating normalization of RBC turnover (Figure 4C). This improvement of RBC survival was further supported by reduced bilirubin levels and lower expression levels of hepatic *Hmox1* in Hbb^th3/+^ mice treated with REGN7999 (Figure 4, D and E).

### REGN7999 improves anaerobic capacity in Hbb^th3/+^ mice.

RBCs of Hbb^th3/+^ mice treated with REGN7999 are healthier than untreated mice. However, it remained unknown whether treatment translates to a functional improvement of RBCs. As REGN7999 treatment led to changes in RBC size and survival, we tested whether there was a functional correlate in terms of oxygen delivery into tissues. In a treadmill exhaustion run (Supplemental Figure 10A), Hbb^th3/+^ mice ran significantly less than WT mice, suggesting that these mice reached exhaustion faster (Figure 4F and Supplemental Figure 10, B and C). Hbb^th3/+^ mice treated with REGN7999 ran a similar distance as WT animals, indicating that the improvement of RBC health translates to improved tissue oxygen delivery and exercise capacity (Figure 4F). C57BL/6 mice injected with REGN7999 showed no difference in running distance compared with isotype-treated C57BL/6 mice (Supplemental Figure 10D).

We further assessed the anaerobic capacity of Hbb^th3/+^ mice and their response to REGN7999 by measuring blood lactate immediately after exhaustion running. In isotype-treated Hbb^th3/+^ mice, exhaustion running resulted in higher serum lactate levels, despite their running less distance than REGN7999-treated Hbb^th3/+^ mice (Figure 4G). This result is in line with the observed improvements in RBC health. Moreover, at baseline, Hbb^th3/+^ mice have elevated blood lactate levels (Supplemental Figure 10E). Hence, we tested whether Hbb^th3/+^ mice have ineffective lactate removal. A lactate tolerance test and measurements of hepatic lactate dehydrogenase (LDH) activity did not show differences between Hbb^th3/+^ mice and WT littermates, indicating that removal of lactate is unaffected (Supplemental Figure 10, F and G). Conversely, LDH activity in erythrocytes is significantly increased in Hbb^th3/+^ mice, suggesting that RBCs of β-thalassemia mice produce more lactate, especially during exhaustion running; inhibition of TMPRSS6 with REGN7999 improves this (Supplemental Figure 10H).

### REGN7999 increases bone mineral density.

**Figure 5:**
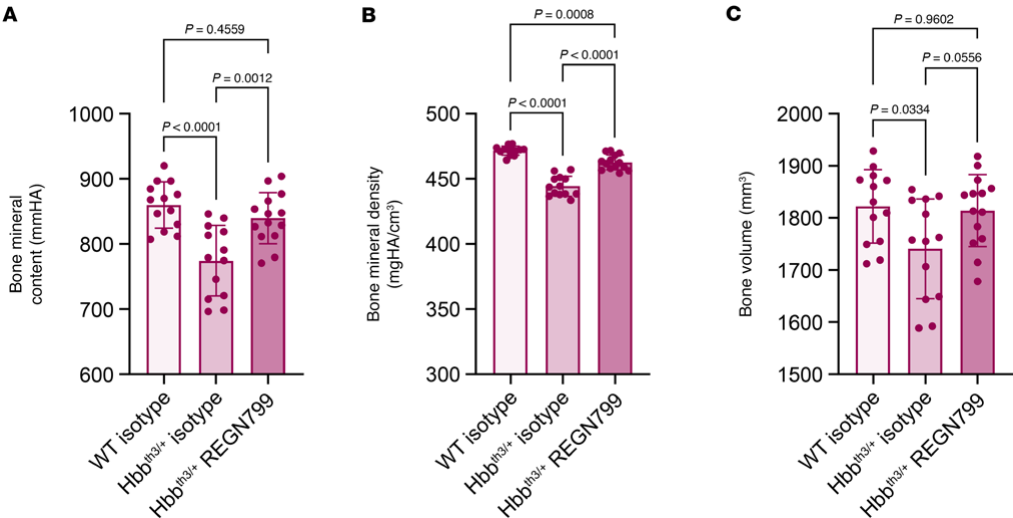
REGN7999 treatment improves bone health in β-thalassemia after 8 weeks of treatment. (**A**–**C**) Bone mineral content, bone mineral density, and total bone volume were measured with μCT in WT or Hbb^th3/+^ mice after treatment with REGN7999 or isotype control. Significance (*P* < 0.05) was determined by 1-way ANOVA with Tukey’s multiple-comparison test.

Secondary to ineffective erythropoiesis and poor bone marrow health, many patients with β-thalassemia experience lower bone quality and have a ~44% higher fracture risk (21, 35). Thus, we tested whether increasing the effectiveness of erythropoiesis and extending the RBC lifespan leads to improvements in bone density by measuring bone mineral density and content as well as bone volume in mice treated with REGN7999 versus isotype-control over an 8-week period. At baseline, Hbb^th3/+^ mice display a significant reduction in bone mineral content, bone mineral density, and bone volume compared with WT littermates. In Hbb^th3/+^ mice injected with REGN7999, bone mineral content and bone mineral density were restored to WT levels, and bone volume was at a similar level of the WT controls yet not significantly different to Hbb^th3/+^ mice treated with isotype control (Figure 5). The improvement in bone quality becomes apparent by 4-weeks after treatment, which coincides with the time that improvements in erythropoiesis are observed (Supplemental Figure 11).

### Compared with Acvr2b(L79D)-Fc, REGN7999 reduces hepatic iron and improves ineffective erythropoiesis.

**Figure 6:**
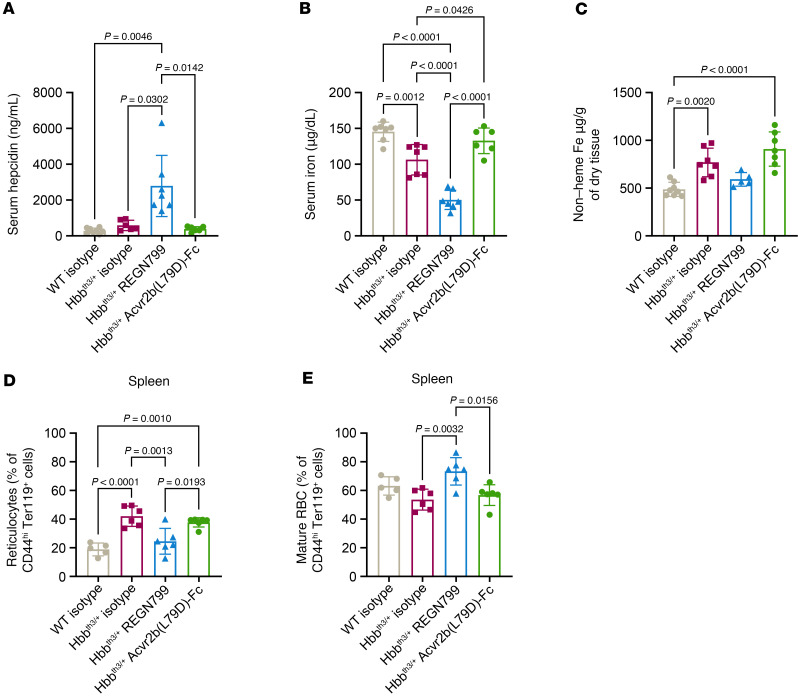
Compared with Acvr2b(L79D)-Fc, REGN7999 treatment reduces liver iron and improves ineffective erythropoiesis after 8 weeks. (**A**–**C**) Serum hepcidin, serum iron, and liver iron content in Hbb^th3/+^ mice or littermate WT controls measured after 8 weeks of treatment of with REGN7999, Acvr2b(L79D)-Fc, or mAb isotype control. (**D** and **E**) Splenic reticulocytes and mature RBCs as measured by flow cytometry in the same animals. Significance was given at *P* < 0.05 and determined by 1-way ANOVA with Tukey’s multiple-comparison test.

We compared the effects of REGN7999 with those obtained with Acvr2b(L79D)-Fc, as it has been reported to improve ineffective erythropoiesis and increase hemoglobin in mice and humans (20, 36–38). Since REGN7999 improves ineffective erythropoiesis and RBC lifespan and health, we performed a comparative analysis between REGN7999 and Acvr2b(L79D)-Fc. After 8 weeks of treatment with either protein, we found that only REGN7999 reduced serum and liver iron (Figure 6, A–C); Acvr2b(L79D)-Fc had no effect on the iron parameters. Furthermore, unlike REGN7999, Acvr2b(L79D)-Fc did not reduce spleen weight (Supplemental Figure 12).

Hematologic analysis shows that, like REGN7999, Acvr2b(L79D)-Fc significantly increases hemoglobin levels and RBC size. However, whereas REGN7999 significantly increases RBC count, Acvr2b(L79D)-Fc does not (Table 2). This difference is also evident by flow cytometry, which shows increased splenic mature RBCs after REGN7999 treatment but not after Acvr2b(L79D)-Fc treatment (Figure 6E). Lastly, reticulocyte counts were only reduced in mice that received REGN7999 (Figure 6D).

### REGN7999 has an acceptable safety profile in healthy human volunteers.

Encouraged by these preclinical data, we tested REGN7999 in healthy human volunteers in a single ascending dose a first-in-human study with 6:2 randomization to REGN7999 or placebo for each of 5 i.v. and 3 s.c. cohorts (clinicaltrials.gov; NCT05481333) (Supplemental Figure 13). In this single-center trial, a total of 64 healthy participants were randomized. Participants were balanced between the REGN7999 and placebo cohorts for age, sex, race, ethnicity, body weight, height, and baseline hematinic laboratory values (Table 3).

The incidence of treatment-emergent adverse effects (TEAE) was similar between the REGN7999 group and placebo group, with 91.7% of participants and 87.5% of participants reporting at least 1 TEAE, respectively (Table 4). None of the healthy participants experienced serious adverse events or deaths or developed symptomatic anemia (Table 4). Notably, there was no evidence of a relationship between dose and incidence of TEAEs in the s.c. or i.v. cohorts. TEAEs were generally mild to moderate in severity, with 2 severe TEAEs (migraine [placebo i.v. in a patient with a history of migraine] and ureterolithiasis [R7999 100 mg i.v. in a patient with a history of urolithiasis]). Both severe TEAEs were not considered related to study drug and resolved by the end of the follow-up period. The most commonly treatment-related adverse effects were headache (41.7% for pooled REGN7999 and 31.3% for pooled placebo), fatigue, nausea, and diarrhea (Table 5).

### Single administrations of REGN7999 increase serum hepcidin and ferritin and decrease serum iron and transferrin saturation.

**Figure 7:**
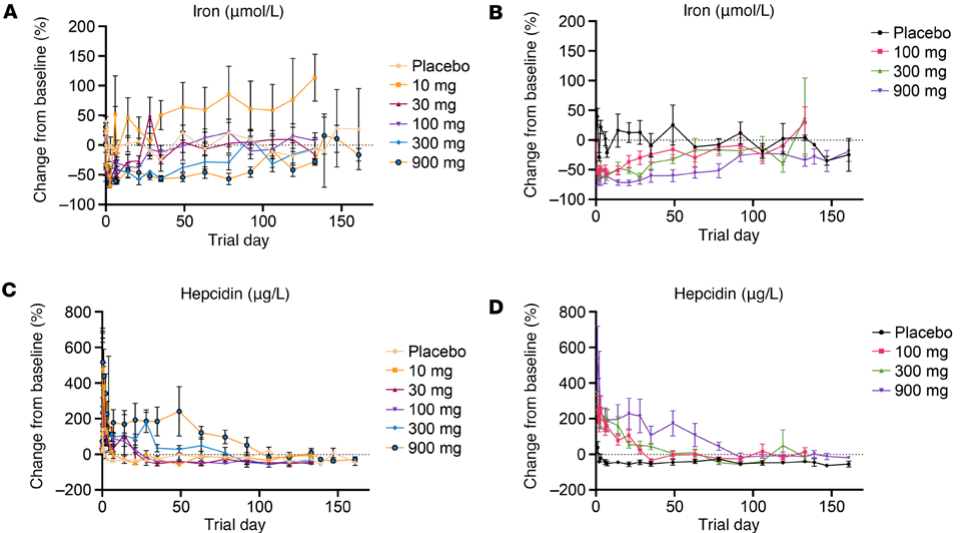
REGN7999 administration reduces serum iron and increases hepcidin in healthy clinical trial participants. (**A** and **B**) In the first-in-human study, REGN7999 reduced serum iron independent of i.v. (**A**) or s.c. (**B**) injection. (**C** and **D**) In contrast, serum hepcidin levels increased in a dose-dependent manner after i.v. (**C**) and s.c. (**D**) injection.

Following single administrations of REGN7999, serum iron acutely decreased from baseline by 54.8%–71.7% in all groups within 1 day following treatment. Return to baseline was dose dependent, independent of route of administration (Figure 7, A and B, and Supplemental Figure 14, A and B).

Hepcidin concentrations in serum showed acute increases in mean percent change from baseline, by 220%–516% in the i.v. cohorts and 259% to 534% in the s.c. cohorts within 4 days following treatment (Figure 7, C and D, and Supplemental Figure 14, C and D). This initial increase was followed by a rapid decrease, with subsequent maintenance of a sustained elevation from baseline with the highest i.v. and highest s.c. doses returning to baseline by day 106 and 92, respectively (Figure 7, C and D). The placebo arms had transient increases (138% and 40% for the placebo i.v. and s.c. cohorts, respectively), followed by stabilization below the baseline value, potentially driven by minor physiologic iron deficiency induced by study blood draws.

**Figure 8:**
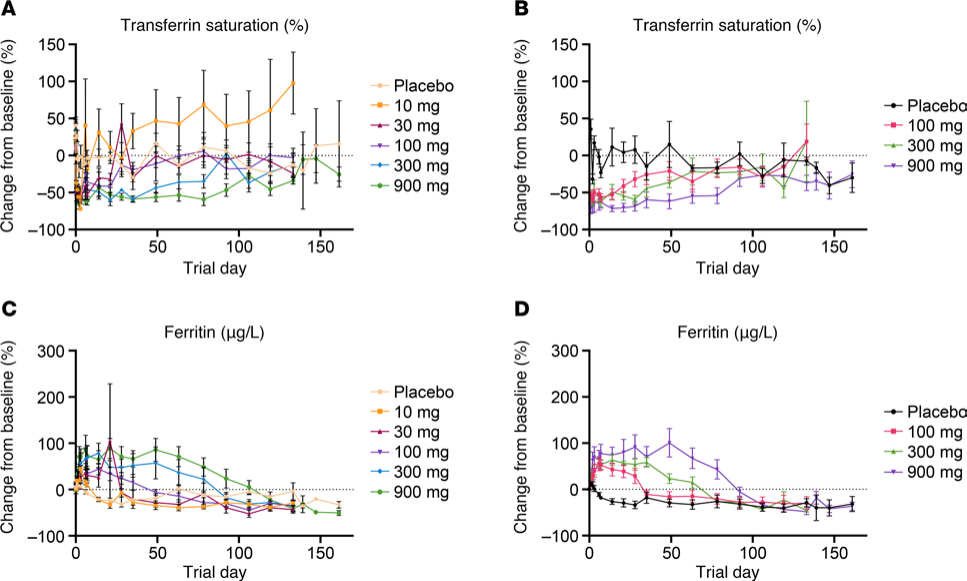
REGN7999 administration reduces transferrin saturation and increases serum ferritin in healthy clinical trial participants. (**A** and **B**) REGN7999 administration in the first-in-human study reduced transferrin saturation independent of i.v. (**A**) or s.c. (**B**) administration. (**C** and **D**) In contrast, serum ferritin levels increased in a dose-dependent manner after i.v. (**C**) and s.c. (**D**) administration.

Transferrin saturation was similarly reduced by REGN7999 treatment, with deep initial reductions within 4 days following treatment, ranging from –50.7% to –72.2% in the REGN7999 i.v. cohorts and –52.6% to –71.8% in the REGN7999 s.c. cohorts, followed by gradual return to baseline (Figure 8 and Supplemental Figure 15, A and B). The 900 mg i.v. REGN7999 cohort and 900 mg s.c. REGN7999 cohort remained below baseline at the cessation of follow-up. The placebo i.v. and s.c. groups had fluctuations in the initial days from administration and maintained higher or at-baseline levels through end-of-study (EOS).

In normal homeostasis and in most iron-deficiency anemia patients, serum ferritin correlates with transferrin saturation, with decreased serum ferritin reflecting reduced intracellular iron stores. Following REGN7999 treatment, however, ferritin concentrations in serum initially increased 19.6%–70.9% in the i.v. cohorts and 40.1%–69% in s.c. cohorts within 4 days following treatment before returning to baseline (Figure 8 and Supplemental Figure 15, C and D). A dose-dependent increase was also observed in the duration of the change from baseline in mean ferritin concentration. In the highest REGN7999 i.v. dose cohorts of 300 mg and 900 mg, mean ferritin concentrations returned to baseline by day 92 and day 107, respectively. In the highest REGN7999 s.c. dose cohorts of 300 mg and 900 mg, mean ferritin concentrations returned to baseline by day 78 and day 92, respectively (Figure 8).

### REGN7999 induces expected changes to erythropoiesis in healthy participants.

Iron deficiency anemia is associated with impaired erythropoiesis that manifests as low reticulocyte counts, low RBC count, decreased reticulocyte and RBC hemoglobin, and microcytosis. Participants exposed to single doses of REGN7999 showed expected changes in erythropoiesis, consistent with the prolonged iron restriction induced by REGN7999 in non-iron-overloaded participants.

Reticulocyte count initially decreased from day 2 through day 8 followed by a return to baseline within 64 days across all REGN i.v. and s.c. dose cohorts, corresponding to recovery of serum iron and transferrin saturation. The maximum mean percent change from baseline occurred at the 900 mg dose level and was –14.8% (day 50) for the 900 mg i.v. cohort and –11.4% (day 36) for the 900 mg s.c. cohort. Similarly, dose-dependent decreases in reticulocyte hemoglobin began within 4 days after REGN7999 administration. In the placebo group, reticulocyte hemoglobin fluctuated within 3% of baseline values.

Decreases in MCV, reticulocyte count, and reticulocyte hemoglobin through EOS were consistent across i.v. and s.c. dose cohorts (Supplemental Figure 16). The MCH fluctuated within the first 22 days followed by a transient dose-dependent decrease below baseline values in i.v. and s.c. REGN7999 dose cohorts (–10.1% and –7.9%, respectively). In the placebo group, there was a modest decrease in MCH with values fluctuating within 2% of baseline.

RBC counts decreased beginning day 4 (from –1.2% to –5.9% in the i.v. cohorts and –1.4% to –6.0% in the s.c. cohorts) and returned to baseline around day 64.

**Figure 9:**
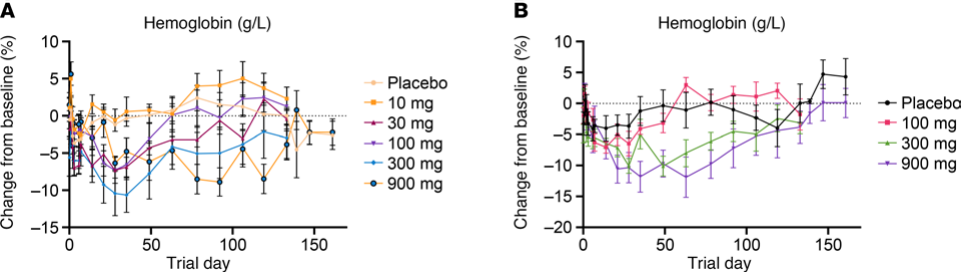
REGN7999 administration transiently reduces hemoglobin in healthy clinical trial participants. (**A** and **B**) REGN7999 administration in the first-in-human study was associated with dose-dependent reductions in hemoglobin in non-iron-overloaded participants, independent of i.v. (**A**) or s.c. (**B**) administration.

In evaluating hemoglobin over time, there were no early discernable treatment differences between REGN7999 i.v. and s.c. doses and placebo (Figure 9). However, the highest i.v. and s.c. REGN7999 doses had late reductions in mean hemoglobin (up to –8.9% in the highest i.v. dose and up to –11.9% in the highest s.c. dose), with the nadir occurring at day 36 for the highest i.v. and day 64 for the highest s.c. dose.

Overall, in healthy volunteers, REGN7999 had an acceptable safety profile and caused rapid increases in serum hepcidin, with corresponding decreases in serum iron and transferrin saturation, though with increases in serum ferritin. This led to expected changes in hematologic parameters consistent with those associated with the development of iron deficiency anemia.

## Discussion

In spite of significant progress in the treatment of β-thalassemia, whether through overall improvements in patient care or, more recently with the advent of gene therapy approaches (39), iron overload remains an important comorbidity (40). Hence, there is still a need for treatments that normalize iron homeostasis in β-thalassemia. Human genetics informs a potential path to the development of such a treatment, as β-thalassemia patients that harbor loss-of-function variants in *TMPRSS6* — which otherwise cause IRIDA (25) — have reduced liver iron (26). This effect is reproduced in Hbb^th3/+^ mice (41). These findings led us to develop human monoclonal antibodies that block TMPRSS6 as a potential therapy for the treatment of iron overload.

Using Hbb^th3/+^ mice, we show that anti-TMPRSS6 mAB REGN7999 reduces liver and serum iron and reduces oxidative stress in RBCs. This improves RBC lifespan, bone health and exercise tolerance, and normalizes spleen size. These favorable effects of REGN7999 on liver iron, erythropoiesis, and physical fitness present a potential improvement over current standard of care. For example, iron chelators target liver iron loading yet do not improve inefficient erythropoiesis and do not ameliorate splenomegaly. Luspatercept improves erythropoiesis and increases hemoglobin levels in β-thalassemia patient without reducing liver iron or splenomegaly (42).

Despite reducing liver iron and improving ineffective erythropoiesis, REGN7999 treatment did not increase hemoglobin levels. This may be due to the low MCV of otherwise healthy RBCs in treated Hbb^th3/+^ mice. We anticipate that reducing the REGN7999 dose might increase hemoglobin incorporation and MCV, but the efficiency of liver iron unloading might be impacted. In clinical studies, it might be important to find a balance between iron unloading and increasing hemoglobin incorporation by titrating the REGN7999 dose accordingly. However, our preclinical data suggest that focusing on hemoglobin increase alone to determine if the treatment is efficacious might not be the ideal strategy, given that we observe significant improvements in RBC health and physical fitness in Hbb^th3/+^ mice despite smaller RBC size.

Previously published preclinical studies suggest potential benefit of reducing TMPRSS6 as an adjunct to the administration of iron chelator deferiprone in Hbb^th3/+^ mice (43). Additionally, the combination of a luspatercept-like drug and TMPRSS6-ASO has demonstrated advantages over treatment with either agent alone in the mouse (44), suggesting that the strategy of blocking TMRPSS6 in combination with one of these drugs could be an attractive approach to treatment of patients with β-thalassemia.

Encouraged by our preclinical studies, we initiated a first-in-human single ascending i.v. or s.c. dose healthy volunteer study (clinicaltrials.gov; NCT05481333). REGN7999 had an acceptable safety profile and resulted in a deep and sustained decrease in circulating iron, particularly at higher doses.

The pattern in serum hepcidin noted after single doses of REGN7999 is characterized by an initial spike, followed by a rapid drop and maintenance of a sustained increase from baseline. This pattern reveals in-human evidence of the relative magnitude of component regulatory pathways involved in hepcidin expression, wherein there are distinct contributions from transferrin-bound iron and BMP signaling (as well as IL-6 signaling). BMP signaling in the liver is in part induced by iron availability, which causes angiocrine BMP2 from hepatic sinusoids, and in part by basal expression of BMP ligands (45). However, the relative contributions of these components to hepcidin expression in humans have not been previously defined.

Inhibition of TMPRSS6 within 8 hours (by i.v. administration of REGN7999) results in induction of hepcidin expression, up to ~5-fold above baseline. However, as a likely result of this abrupt increase in serum hepcidin, there is a profound decrease in circulating transferrin-bound iron. We attribute this to reticuloendothelial sequestration, as all participants were fasting prior to the administration of study drug, to minimize the effect of gut iron on early pharmacodynamic markers. Following the rapid decline of transferrin-bound iron, the component of hepcidin expression induced by transferrin/transferrin-receptor signaling is likely abrogated, resulting in the sudden drop in hepcidin levels. However, this does not result in an immediate return to baseline — rather, hepcidin is subsequently maintained ~2-fold above baseline (at the highest doses of REGN7999), likely due to maintenance of the basal BMP signaling component.

Our data show that REGN7999 recapitulates effects on hepcidin and serum iron previously described with TMPRSS6-ASO or siRNA, although the magnitude of serum iron reduction in our study exceeds the reduction achieved using ASO (27) or siRNA. Ferroportin inhibiting molecules have additionally been described to decrease serum iron in healthy volunteers, with a magnitude of reduction similar to what was achieved in this study, although with transient duration of action.

In summary, we show in preclinical studies that inhibiting TMPRSS6 with a potent antibody improves clinically relevant sequalae of disease such as liver iron loading, splenomegaly, bone, and RBC health. In a phase I study, we show that REGN7999 significantly increases hepcidin and dramatically reduces serum iron for up to 12 weeks, with an acceptable tolerability profile. Due both to its potency versus other biologic agents, and the prolonged and consistent nature of its pharmacologic effects versus small molecule ferroportin inhibitors, REGN7999 is under further clinical development as a therapeutic for the treatment of iron overload in patients with non-transfusion-dependent β-thalassemia (NCT06421636) and other hematologic diseases.

## Methods

Supplemental Methods are available online with this article.

### Sex as a biological variable.

Our study examined male and female mice, preadolescent female monkeys, and male and female healthy human volunteers.

### Monoclonal antibody (mAb) and cell line generation.

REGN7999 (Regeneron Pharmaceuticals Inc.) is a human mAb generated by VelocImmune mice (46, 47) using human TMPRSS6 DNA, with an IgG4 constant domain. VelocImmune mice are genetically modified to produce antibodies with human variable regions, which enables efficient selection and development of human antibodies.

To select candidate antibodies, we used several screening methods, including cell binding and substrate cleavage assays, ELISA, and flow cytometry to identify cell-surface HJV expression. To identify where REGN7999 binds TMPRSS6, we used Cryo-EM. These methods are described in detail in the Supplemental Methods, and in the patent for REGN7999 (US20220411487A1).

### Mouse studies.

We injected REGN7999, Acvr2b(L79D)-Fc (produced in-house), and REGN1945 isotype controls s.c. for up to 12 weeks (10 mg/kg weekly; in the first week the mice received 2 injections at days 0 and 3). Mice (male/female) were housed in a controlled environment (12-hour light/dark cycle, 60%–70% humidity, 22°C ± 1°C) and fed ad libitum a normal chow diet containing 240 ppm of iron (PicoLab Rodent Diet, LabDiets). We used Hbb^th3/+^ mice, since these animals display increased reticulocyte count, low RBC count, and low hemoglobin and show a significant liver iron overload (32).

### Cynomolgus monkey studies.

The NHP study was conducted by a contract research organization that is accredited by the Association for the Assessment and Accreditation of Laboratory Animal Care and was performed in compliance with the Animal Welfare Act, the Guide for the Care and Use of Laboratory Animals, and the Office of Laboratory Animal Welfare.

### Serum iron measurements and hematology.

Serum iron and total iron-binding capacity to calculate iron saturation were measured using Siemens ADVIA Chemistry XPT. Hematologic parameters were obtained using the Oxford Science GENESIS Hematology System.

### Determination of serum hepcidin.

Serum hepcidin was measured using a commercially available ELISA kit (Intrinsic LifeSciences, HMC-001) according to the manufacturer’s protocol.

### Liver iron content.

Liver iron content was measured using 2 methods. The first utilized a histological method (Perl’s stain), modified after P.J. Brundelet (48). The second used a biochemical method developed by Torrance and Bothwell (49).

### Flow cytometry to measure RBC differentiation.

Isolated single RBC suspensions from spleen or bone marrow were incubated with mouse Fc-blocker (anti-CD16/32, catalog 553142, BD Biosciences) for 10 minutes followed by incubation with FITC anti-Ter119 (catalog 557915), phycoerythrin anti-CD71 (catalog 567206), allophycocyanin (APC) anti-CD44 (catalog 559250), and an APC-cyanine7 anti-CD45 (catalog 557859)/CD11b (catalog 557657)/Ly6G cocktail (catalog 560800) (all antibodies BD Biosciences) for 30 minutes. Dead cells were identified by LIVE/DEAD fixable stain (Thermo Fisher Scientific) and excluded.

### Measurement of RBC oxidative stress and senescence.

To identify reactive oxygen species and annexin V display in RBCs, staining was performed with 10 μM 6-carboxy-2’,7’-dichlorodihydrofluorescein diacetate (carboxy-H2DCDFDA; Thermo Fisher Scientific) to measure oxidative stress or with an annexin V^+^ staining kit (BioLegend) to detect senescence.

### Measurement of RBC turnover.

Mice were injected retro-orbitally with 50 mg of NHS-PEG4-biotin (Pierce, Thermo Fisher Scientific) for 3 consecutive days. Approximately 10 μL of blood was collected from the tail vein and incubated with FITC anti-Ter119 (catalog 557915) and APC-streptadivin (catalog 554067) (both BD Bioscience) 1 day after the last biotin injection. To calculate RBC turnover, the fractions of biotin-labeled RBCs were analyzed using flow cytometry (Cytoflex LX, BD Biosciences).

### Bone density measurements.

Mouse whole-body composition analysis was assessed using in vivo μCT (Quantum GX, PerkinElmer). Briefly, mice were anesthetized by isoflurane and the whole body except the head was scanned, with a field of view at 60 mm × 120 mm. The x-ray source was set to a current of 88 μA and a voltage of 90 kVp, with a voxel size of 240 mm. The CT imaging was visualized and quantified using Vivaquant software (VivaQuant). Whole-body bone was auto segmented by threshold, and then bone volumes, bone mineral density, and bone mineral content were calculated.

### REGN7999 in a first-in-human study.

A phase I, double-blind, placebo-controlled, single–ascending dose study was designed to assess the safety, tolerability, and pharmacodynamics/pharmacokinetics of REGN7999 in healthy adults. Participants were healthy adults at a single center (clinicaltrials.gov; NCT05481333). Key inclusion criteria included hemoglobin, hematocrit, serum iron, and transferrin saturation at or above the lower limit of normal for age and sex (Supplemental Table 1). Female participants were required to be nonmenstruating. The study was conducted in accordance with the ethical principles originating from the Declaration of Helsinki and was consistent with the International Conference on Harmonization/Good Clinical Practices and applicable regulatory requirements. All human research was approved by health authorities and institutional review boards, and all participants provided written informed consent.

The primary objective of the study was to identify the safety and tolerability of single doses of REGN7999. Secondary and exploratory objectives included characterizing the drug-concentration profile of REGN7999 and evaluating the effects of REGN7999 on serum biomarkers of iron homeostasis, respectively. The data cutoff date was September 21, 2023.

Participants were randomized 6:2 to receive REGN7999 or placebo in 5 ascending i.v. and 3 ascending s.c. cohorts.

### Clinical trial data collection and measurements.

To minimize the circadian and dietary influence on hepcidin and iron biomarkers, participants were required to be fasting overnight prior to baseline lab draws. Following administration of REGN7999 or placebo on study day 1, participants were admitted overnight for observation and maintained on an iron-restricted diet (no red meat, no iron-fortified foods). For subsequent study visits, participants were required to fast overnight prior to lab draws. Participants were followed for up to 26 weeks following the administration of REGN7999 of placebo.

### Statistics.

Data from mouse studies are presented as mean ± SD. Depending on the experiment, either a 1-way or 2-way ANOVA with Tukey post hoc test for multiple comparison or a Student’s *t* test was performed. Depending on the expectation of the outcome, a 2-tailed or 1-tailed t-test was utilized. When *P* values were < 0.05, the outcome was considered statistically significant.

### Study approval.

All procedures related to mouse studies were approved by the Institutional Animal Care and Use Committee (IACUC) and conducted in compliance with approved protocols. The nonhuman primate study was conducted by a contract research organization that is accredited by the Association for the Assessment and Accreditation of Laboratory Animal Care and was performed in compliance with the Animal Welfare Act, the Guide for the Care and Use of Laboratory Animals, and the Office of Laboratory Animal Welfare. All studies were approved by an IACUC.

The human study was conducted in accordance with the ethical principles originating from the Declaration of Helsinki and was consistent with the International Conference on Harmonization/Good Clinical Practices and applicable regulatory requirements. All human research was approved by health authorities and IRBs, and all participants provided written informed consent. All animals were preadolescent female animals.

### Data availability.

Qualified researchers may request access to study documents (including the clinical study report, study protocol with any amendments, blank case report form, and statistical analysis plan) that support the methods and findings reported in this manuscript. Individual anonymized participant data will be considered for sharing once the product and indication has been approved by major health authorities (e.g., FDA, European Medicines Agency’ [EMA], Pharmaceuticals and Medical Devices Agency [PMDA]), if there is legal authority to share the data and there is not a reasonable likelihood of participant reidentification. Submit requests to https://vivli.org/ The cryoEM map and atomic coordinates of TMPRSS6 REGN7999 Fab/REGN8023 Fab complex has been deposited into the Protein Data Bank and the Electron Microscopy Data Bank using accession codes 9NRC and 49728, respectively. Values for all data points in graphs are reported in the Supporting Data Values file.

## Author contributions

HEL, LI, BC, HJK, LK, NMD, YR, JHK, EK, JJF, MP, LSN, KS, MF, and KCN performed the experiments and analyzed the results. HEL, JJF, KCN, VI, ANE, and SJH designed the preclinical research. HEL, HFC, and MWR designed, coordinated, and analyzed the NHP studies. KM, NS, SR, ELT, HEH, LF, MEB, OAH, LP, and GVL designed, coordinated, and analyzed the clinical research. SR and GVL performed the clinical research. WO and AJM advised on research strategy and antibody production. HEL, ANE, and SJH created the figures and wrote the manuscript.

## Supplementary Material

Supplemental data

Supporting data values

## Figures and Tables

**Table 1:**
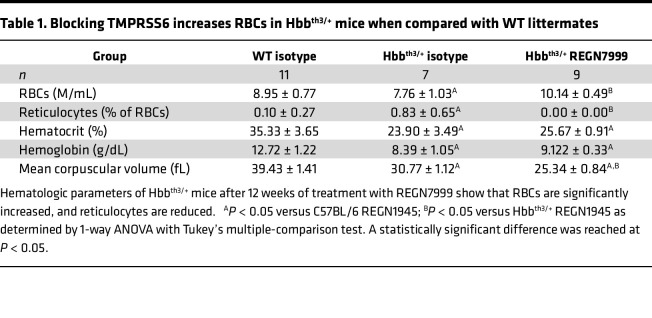
Blocking TMPRSS6 increases RBCs in Hbb^th3/+^ mice when compared with WT littermates

**Table 2:**
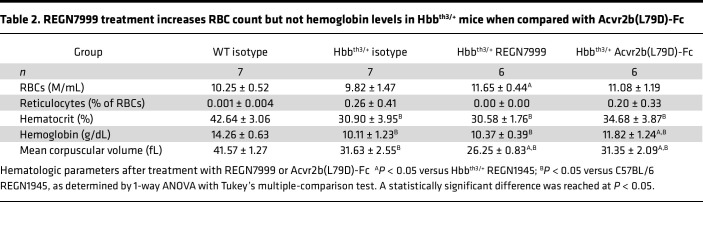
REGN7999 treatment increases RBC count but not hemoglobin levels in Hbb^th3/+^ mice when compared with Acvr2b(L79D)-Fc

**Table 3:**
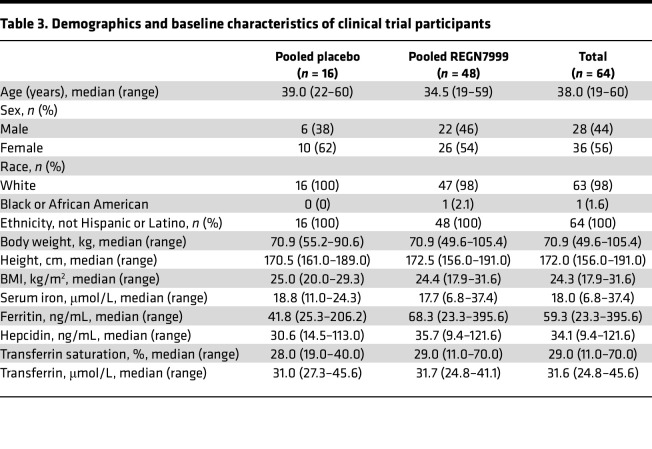
Demographics and baseline characteristics of clinical trial participants

**Table 4:**
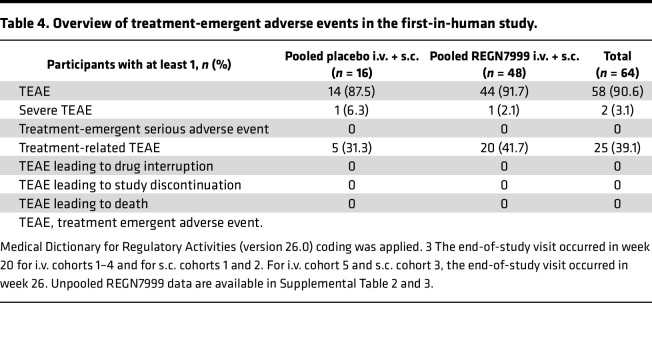
Overview of treatment-emergent adverse events in the first-in-human study.

**Table 5:**
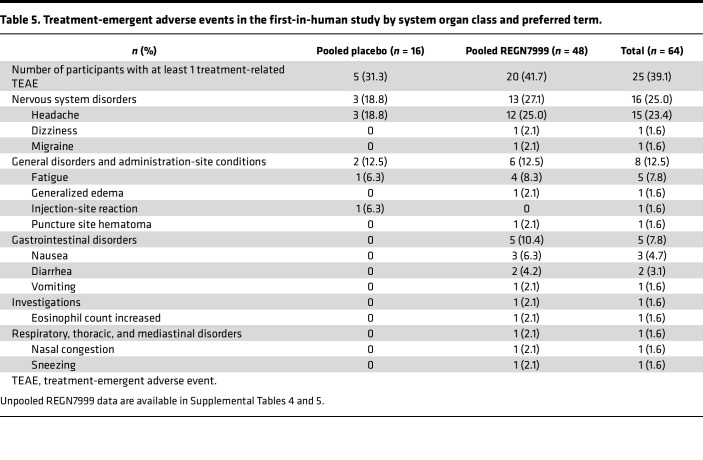
Treatment-emergent adverse events in the first-in-human study by system organ class and preferred term.

## Version 1 06/23/2025

Electronic publication
