# Supplementary material for: A TMPRSS6-inhibiting mAb improves disease in a β-thalassemia mouse model and reduces iron in healthy humans
Source: JCI Insight. 2025 Jun 23;10(12):e191813. doi: 10.1172/jci.insight.191813 (PMC12220967; doi:10.1172/jci.insight.191813)
Supplement: Supplemental data [file jciinsight-10-191813-s196.pdf]

Supplemental methods

Monoclonal antibody (mAb) and cell line generation

REGN7999 was created by immunizing Regeneron Pharmaceutical Inc.'s VelocImmune® mice (1, 2) using human TMPRSS6 DNA. The VelocImmune platform uses mice that are genetically modified to produce mAbs with human variable regions, and enables efficient selection, and development of human Abs. The mAb has an IgG4 constant domain, and is fully human, monoclonal, and specific to TMPRSS6.

For the selection of the Abs, we generated HEK293 cells overexpressing full-length human hemojuvelin (HJV) and human reference (hTMPRSS6), human variant (hTMPRSS6 - K253E, hTMPRSS6 - P555S, and hTMPRSS6 - V736A), monkey (mfTMPRSS6), or mouse (mTMPRSS6) TMPRSS6. Additional cell lines were generated expressing hTMPRSS6 and mTMPRSS6 with a c-terminal triple Myc-tag. Stably expressing cells were selected with antibiotics. Cell lines expressing Myc-tagged TMPRSS6 were sorted for high TMPRSS6-expressing populations using an anti-Myc Ab, and the non-Myc-tagged mouse TMPRSS6 cells were sorted using a TMPRSS6 Ab.

For cell-binding studies and fluorogenic substrate cleavage assays, we used HEK293 cells overexpressing only TMPRSS6 forms without human HJV. Parental HEK293 cells were used as a control in all experiments. All cell lines were cultured with 500 mg/mL of G418 and 100 mg/mL hygromycin B in DMEM containing 10% (v/v) FBS, 100 units of penicillin, 100 mg of streptomycin, and 292 mg/ml of L-glutamine.

Cryogenic electron microscopy (cryoEM) sample preparation and data collection

Purified human TMPRSS6 ectodomain protein (residues G77-T811 with catalytic inactivating mutation S762A, fused to a C-terminal Myc-Myc-6x histidine tag) was mixed with enzymatically prepared Fab fragments of REGN7999 and REGN8023 (a non-competing antibody added to facilitate cryoEM structure determination) and incubated for 30 minutes on ice to form TMPRSS6-Fab-Fab complex. The complex was purified using an SEC column (Superdex 200 Increase 10/300 GL, Cytiva) connected to an AKTA Avant 25 chromatography system (Cytiva). SEC running buffer contained 25 mM Tris-HCl pH 7.5, 150 mM NaCl.

Freshly purified TMPRSS6-Fab-Fab complex at a concentration of ~1.75 mg/mL was mixed with ~60 μM n-Dodecyl-β-D-maltopyranoside (Anatrace) immediately before pipetting 3.5 μL of the mixture onto a UltrAufoil R1.2/1.3, 300 mesh grid (Quantifoil). Excess liquid was blotted away using filter paper and the grid was plunge frozen into liquid ethane cooled by liquid nitrogen using a Vitrobot Mark IV (ThermoFisher) operated at 4° C and 100% humidity. The grid was then inserted into a Titan Krios G3i microscope (ThermoFisher) equipped with a K3 BioQuantum imaging system (Gatan). 3,580 movies were collected in counted mode at a nominal magnification of 105,000x (0.85 Å pixel size) using EPU software. Each movie contained 46 dose fractions over a 2 second exposure, and the total acquired dose per $Å^2$ was ~40 electrons (**Supplemental Table 6**).

CryoEM data processing and map generation

Initial processing of cryoEM data using Cryosparc v2.14.2 (3) resulted in a 3.4 Å resolution reconstruction of the TMPRSS6/REGN7999 Fab/REGN8023 Fab complex. The map

used for model building was calculated using a Relion version 3.1 (4) single particle processing pipeline. Movies were motion-corrected using Relion's implementation of MotionCor2 and CTF parameters were estimated using gctf (5) 3,411 of 3,580 total micrographs were selected for further processing based on having a CTF resolution estimation of better than 4 Å. 1,445,534 particles were picked from the micrographs using 2D template-based autopicking and extracted as 3x binned particle images (2.55 Å per pixel). Three rounds of 2D classification were conducted to remove false positives and broken or incomplete complexes, resulting in 1,034,494 particles. These particles were subjected to 3D classification requesting 8 classes, using an initial reference model calculated in CryoSparc. 270,021 particles from the two best classes corresponding to TMPRSS6/REGN7999 Fab/REGN8023 Fab complex were re-extracted as unbinned particle images and subjected to 3D refinement, which produced a 3.4 Å resolution map in which the Fabs and catalytic domains were well resolved but the remaining N-terminal domains of the TMPRSS6 were poorly resolved. To better resolve the N-terminal domains, focused classification was carried out without alignment, applying a soft mask that excluded the Fabs and catalytic domain. A single class containing 146,035 particles was identified as having improved density for the N-terminal domains. Refinement of these particles resulted in a 3.6 Å resolution map. Two rounds of CtfRefine and Bayesian polishing were followed by three additional focused 3D classification runs without alignment in which soft masks surrounding the CUB and SEA domains were applied, resulting in a final set of 105,142 particles. These particles were then refined into a 3.29 Å resolution (FSC=0.143) map of the TMPRSS6/REGN7999 Fab/REGN8023 Fab

complex with improved features in N-terminal domains. For model building, the map was filtered to its local resolution values and sharpened to a B factor of -76 Å^2 in Relion.

CryoEM structure building and refinement

Manual model building was carried out using Coot version 0.8.9 (6) and real space refinements were conducted in Phenix versions 1.17 and 1.21 (7). An initial model for human TMPRSS6 was obtained from the AlphaFold database (8, 9). Homology models generated from an internal Fab structure were used as initial models for REGN7999 and REGN8023 Fab. Following docking of the starting models into the map, multiple iterations of manual adjustment in Coot followed by real space refinement in Phenix were conducted to generate the final structure. The model for TMPRSS6 encompasses residues 82-811, excepting residues 573-580 and 755-756, which are in apparently disordered loops. Coordinates for the SEA domain (residues 82-208) were truncated to poly-alanine due to weakly resolved density. N-linked glycan residues were built where the map showed clear density (N136, N216, N338, N433, N453, N518). Ca^{2+} ions were built into strong map signals at the center of conserved Ca^{2+}-binding acidic clusters in each of the three LDLRA domains. Structure validation statistics were monitored using Molprobity (10). Structural data were analyzed and visualized using Pymol 2.5.4 (11), Chimerax 1.2.5 (12), and Chimera 1.18 (13).

Cell binding assays

Binding of REGN7999 to TMPRSS6 was evaluated in HEK293 cells expressing different TMPRSS6 variants (see above). Cells were collected from plates using an enzyme-free dissociation buffer and resuspended in PBS + 2% FBS before adding 5 x 10^5 cells to each well. REGN7999 was added to wells at concentrations ranging from 6.1 pM to 100 nM, and incubated at 4°C for 30 minutes. Cells were then washed and incubated with 4 µg/mL of Alexa Fluor 647-conjugated anti-human secondary mAb at 4°C for 30 min. Samples were filtered and analyzed on the IquePlus cytometer (Sartorius, Bohemia, NY, USA). MFI was calculated by Forecyt Software (Sartorius, Bohemia, NY, USA) and plotted in Prism software (GraphPad, Boston, MA, USA) using a 4-parameter analysis to obtain EC_{50} values. A 'secondary-only point' of the average MFI of cells treated with secondary mAb only from the day of the experiment was included on the graph but excluded from the EC_{50} calculation.

Fluorogenic substrate cleavage assay

Inhibition of TMPRSS6 proteolytic activity was evaluated in HEK293 cells expressing different TMPRSS6 versions (see above). Poly-D-lysine coated plates were prepared with 1 x 10^5 cells/well, and incubated overnight at 37°C. The next day, REGN7999 or isotype control mAb were added with a concentration range of 16.9 pM to 1000 nM for 30 min at 25°C. Cells were then incubated at 37°C for 60 min with the fluorogenic peptide substrate Boc-Gln-Ala-Arg-AMC (Enzo Life Sciences Inc., Long Island, NY, USA). Fluorescence was recorded in relative fluorescence units (RFUs) using a plate reader at 380 nm excitation and 460 nm emission. RFU values were plotted in

GraphPad Prism to obtain IC_{50} values. A 'zero point' containing only cells ($RFU_{cells\ alone}$) and no mAb was included on the graph but excluded from the IC_{50} calculation.

Maximum percent inhibition was calculated from the observed RFU values using the following equation:

$$Maximum\ percent\ inhibition = \frac{\mathrm{RFU_{cells\ alone}} - \mathrm{RFU_{experimental}}}{\mathrm{RFU_{cells\ alone}} - \mathrm{RFU_{HEK293\ parental}}} \times 100$$

In this equation, $RFU_{cells\ alone}$ is the fluorescence value recorded without mAb (ie, the baseline level of proteolytic activity for the tested HJV- and TMPRSS6-expressing cell line). $RFU_{experimental}$ is the fluorescence value recorded across the tested range of mAb concentrations. $RFU_{HEK293\ parental}$ is the average fluorescence value observed for the isotype control mAb at all concentrations tested with parental HEK293 cells. Maximum percent inhibition was calculated by averaging the 2 highest percent inhibition values across the tested range of mAb concentrations.

ELISA measurement of HJV

To calculate the inhibition of TMPRSS6 activity by REGN7999, we measured cleaved HJV in supernatant. The assay cell lines were plated in flat-bottomed 96-well plates and REGN7999, protease inhibitor aprotinin (Sigma), or isotype control were added to each well and incubated overnight at 37°C. The following day, supernatants were harvested and an ELISA was performed using human RGM-C/Hemojuvelin DuoSet ELISA kit

(R&D Systems, Minneapolis, MN, USA) according to the manufacturer's protocol. Optical density was obtained using SpectraMax i3 (Molecular Devices, San Jose, CA, USA), and results were analyzed with Prism software (GraphPad) to obtain IC_{50} values. The inhibition percentage was calculated with the following equation:

$$Inhibition, [\%] = \frac{Soluble\ HJV\ baseline - Soluble\ HJV\ Ab}{Soluble\ HJV\ Baseline - Soluble\ HJV\ protease\ inhibitor} \times 100$$

Soluble HJV baseline indicates the amount of soluble HJV from the supernatant of HJV-TMPRSS6 over-expression cells in the isotype control-treated group. Soluble HJV REGN7999 is the amount of soluble HJV from the maximum concentration of Ab-treated assay samples, and soluble HJV protease inhibitor is the amount of soluble HJV from the serine protease inhibitor (aprotinin)-treated sample.

Flow cytometry to identify increased HJV expression in response to REGN7999 treatment

HEK293.hHJV/3xMyc.hTMPRSS6 and HEK293.hHJV/3xMyc.mTMPRSS6 cell lines were incubated overnight with 50 nM of REGN7999. The following day, cells were harvested for surface staining. To stain the cell surface of human HJV, cells were blocked with FcR-blocker (Miltenyi Biotech, Bergheim, Germany) and stained with an anti-HJV antibody (Abcam, Cambridge, UK; 1:400, on ice, 1 h) followed by incubation with a donkey anti-mouse IgG AF647 antibody (Invitrogen, Waltham, MA, USA; 1:2000, in ice, 40 min). The stained cells were measured using an Accuri C6 flow cytometer (Becton Dickenson, Franklin Lakes, NJ, USA). A serine protease inhibitor, aprotinin (Sigma, 2 μg/mL), and a protease inhibitor cocktail (Sigma, 1:200) were included as controls to block protease activity in HEK293.hHJV/3xMyc.h/mTMPRSS6 cells.

Mouse studies

Initially, we tested the mAb in mice that, for reasons not related to the experiments, expressed humanized HJV on a C57Bl/6 background (HJV^{Humin}). These mice were not physiologically different from C57Bl/6 mice and were used as WT controls for some studies. Animals were bled 7 days before and on the day of injection (day 0) with REGN7999 or isotype control (both 5 mg/kg). The animals were euthanized at day 3. In long-term studies we used either C57Bl/6 mice (Charles River, Wilmington, MA, USA) or $Hbb^{th3/+}$ (strain: $Hbb^{b1TM1Unc1}$ $Hbb^{b2TM1Unc/J}$; and WT littermates; acquired from Jackson Laboratories, Bar Harbor, ME, USA). In some experiments we used male mice, and in other experiments female mice, which is indicated in the figures. All procedures were approved by the Institutional Animal Care and Use Committee and conducted in compliance with approved protocols.

Cynomolgus monkey studies

The non-human primate study was conducted by a contract research organization which is fully accredited by the Association for the Assessment and Accreditation of Laboratory Animal Care. The study was conducted in compliance with the Animal Welfare Act, the Guide for the Care and Use of Laboratory Animals, and the Office of Laboratory Animal Welfare. The study was conducted according to a protocol and procedures approved by an Institutional Animal Care and Use Committee. All animals were pre-adolescent females. REGN7999 and isotype control mAb were administered by i.v. slow bolus injection over a target period of 2–3 min via a cephalic or saphenous vein once on day 1. Blood was collected before injection (pre-dose) and at several time points over a period of 6 weeks to measure serum iron. Serum iron and total

iron-binding capacity were measured by the contract research organization, and the data was analyzed at Regeneron Pharmaceuticals, Inc.

Serum iron measurements

Serum iron was measured using the Siemens ADVIA Chemistry XPT device (Munich, Germany). This device is an FDA-approved clinical analyzer, which is maintained and operated according to the manufacturer's guidelines; the ADVIA chemistry iron assay is based on the work of Artiss et al (14, 15).

Hematology

Hematologic parameters were obtained by using the Oxford Science GENESIS Hematology System (Oxford, CT, USA).

Determination of serum hepcidin in mouse and Cynomolgus monkey

Serum hepcidin was measured using a commercially available ELISA kit (Intrinsic Lifesciences HMC-001 and PHC-001, San Diego, CA, USA) according to the manufacturer's protocol.

Real time-PCR analysis

Tissues/cells were homogenized in TRIzol, and chloroform was used for phase separation. The aqueous phase containing total RNA was purified using the MagMAX™-96 for Microarrays Total RNA Isolation Kit (Ambion by Life Technologies, Carlsbad, CA, USA) according to the manufacturer's specifications. Genomic DNA was removed using the RNase-Free DNase Set (Qiagen, Hilden, Germany). For housekeeper genes we used GAPDH and β-actin. After confirming that none of these

housekeepers were regulated by disease or treatment, we normalized gene expression to GAPDH. All mRNA expression data are presented as ΔC; all primers used are commercially available.

Liver iron content

First tissues for Perl's stain were formalin-fixed for 24 h before being stored in 70% ethanol. After embedding in paraffin, the samples were sent to a contract research organization (Reveal Biosciences, San Diego, CA, USA) to perform Perl's stain including a hematoxylin and eosin counterstain (modified after P.J. Brundelet) (16). Samples were analyzed blinded using the HALO software package.

Next, we measured liver iron based on the method developed by Torrance and Bothwell (17). In brief, liver tissue (<100 mg) was dried at 42°C for 72 h and then submerged in 10% trichloroacetic acid and 10% hydrochloric acid for another 48 h. Then we added 10 µL ddH_2O to 10 µL of the dissolved tissue in a 96-well plate (clear bottom), and add 100 µL of coloring solution (containing thioglycolic acid, sodium acetate, and bathophenanthroline-disulfonic acid). After 10 min at room temperature, extinction was measured at 535 nm.

RBC isolation from spleen, bone marrow, and whole blood

To isolate mature and precursor RBC from the spleen, tissue was minced and filtered through a 70 µm mesh into 10 mL ice-cold buffer (autoMACS buffer containing EDTA and 10% BSA, Miltenyi Biotec, Bergheim, Germany). Bone marrow was collected by cleaning 2 femurs, cutting the hip end open and placing the bones with the open end towards the bottom of a pipette tip in a reagent tube that contained 100 µL of autoMACS

buffer. The tubes were spun briefly in a table-top centrifuge in order to pull the bone marrow out of the bone into the buffer. After this, the reagent tubes were filled to 1 mL with autoMACS buffer. To isolate RBCs from whole blood we used 50 µL of whole blood and added 950 µL autoMACS buffer to the sample.

All samples (spleen/bone marrow/whole blood) were spun at 400 x g, 4°C for 10 min, and 5 x 10^8 cells were incubated in 450 µL autoMACS buffer with 50 µL anti-mouse CD45 magnetic beads (Miltenyi Biotec Bergheim, Germany) for 20 min on ice. Afterwards, cells were magnetically separated on LS columns (Miltenyi Biotec Bergheim, Germany). The flow-through containing the enriched mature and pre-curser RBCs was used for further processing (see below).

Flow cytometry to measure RBC differentiation

All staining procedures were performed on ice. Compensation controls were performed using OneComp Beads (for antibodies) (eBiosciences, Thermo Fisher Scientific, Carlsbad, CA, USA) and ArC™ amine reactive beads (for LIVE/DEAD™ stain) (Molecular Probes, Thermo Fisher Scientific, Carlsbad, CA, USA) and prepared at the time of cell staining. A –1 control panel was prepared for correct gating of target populations. All samples were measured on a CytoFLEX flow cytometer (Beckman-Coulter, Jersey City, NJ, USA).

Measurement of RBC oxidative stress

To identify reactive oxygen species in RBCs, we stained single-cell suspensions of whole blood, spleens, and bone marrow with APC-Cy7 anti-Ter-119, PE anti-CD71 (all Becton Dickenson, Franklin Lakes, NJ, USA) for 30 min at 4ºC. This was followed by a

LIVE/DEAD stain (30 min, 4ºC, aqua-fluorescent, Thermo Fisher Scientific, Carlsbad, CA, USA). After this, we stained the cells with 10 μM 6-carboxy-2', 7'-dichlorodihydrofluorescin-diacetate (Thermo Fisher Scientific, Carlsbad, CA, USA) for 30 min at room temperature. All samples were measured directly after the last step on a CytoFLEX Flow Cytometer (Beckman-Coulter, Jersey City, NJ, USA).

Identifying RBC senescence

Annexin V^+ staining (Biolegend, San Diego, CA, USA) was used as a read-out for RBC senescence. Whole blood, spleen, and bone marrow were prepared as described above and cells were stained with APC-Cy7 anti-Ter-119 (Becton Dickenson, Franklin Lakes, NJ, USA) and PE anti-CD71 (Beckton Dickenson Franklin Lakes, NJ, USA , 30 min, 4ºC) followed by the staining procedure for Annexin V according to the manufacturer's protocol (FITC Annexin V apoptosis kit, Biolegend, San Diego, CA, USA).

Measurement of RBC turnover

Male $Hbb^{th3/+}$ and WT littermates (8–12 weeks of age) were treated with isotype controls or anti-TMRPSS6 for 8 weeks. Four weeks after the start of treatment, we injected the mice retro-orbitally with 50 mg of NHS-PEG4-Biotin (Pierce, Thermo Fisher Scientific, Carlsbad, CA, USA) for 3 consecutive days. One day after the last injection we collected ~10 μL of blood from the tail vein and incubated it with FITC-anti-Ter119 and APC-streptadivin (both Beckton Dickenson Bioscience, Franklin Lakes, NJ, USA) for 30 min. We analyzed the fractions of biotin-labeled RBCs using flow cytometry (Cytoflex LX, Becton Dickenson, Franklin Lakes, NJ, USA) for 4 weeks after the initial biotin injection.

We analyzed the data with the FlowJo software (FlowJo, LLC, version 10, Franklin Lakes, NJ, USA).

Forced running studies

Prior to all experiments, mice were trained for 2 weeks on the belt of a 6-lane motorized treadmill (Series 8 Treadmill, IITC Life Science, Woodland Hills, CA, USA) supplied with shocker plates. For the exhaustion run we increased the base speed of 10 m/min every 5 min by 5 m/min. The maximum amount of time was determined at 60 min. Mice that either remained inactive on the shocker unit or received 500 shocks were removed from the treadmill.

Lactate measurements and lactate tolerance test

To determine lactate production in β-thalassemia mice, we collected 15 µL of blood from the tail vein after the exhaustion run. For the lactate tolerance tests we injected lactate (2 g/kg body weight, i.p.) and collected blood from the tail vein at 0, 15, 30, 60, 90 and 120 min after the injection. Lactate levels were determined by using a lactometer (Nova Biomedical, Waltham, MA, USA) as previously described (18).

Lactate dehydrogenase activity measurements

Lactate dehydrogenase activity was measured using a kit from BioVision (Milpates, CA USA; Cat: K762). Sample preparation, assay, and analysis were performed according to the manufacturer’s protocol.

Measurement of spleen volume

Spleen volume was measured *in vivo* using 2D multislice MRI images of the torso. T2W images were acquired using a Bruker 7T MRI with a T2 RARE sequence ($TE_{(eff)}$/TR = 30/2516.5 ms; TE = 7.5 ms, echo train length = 8; 4 averages; 100 x 100 μm in plane resolution with 1 mm slice thickness; 35 x 35 mm FOV with a 350 x 350 matrix). Regions of interest for volume measurements were drawn manually using VivoQuant software (Invicro, Needham, MA, USA)

Supplemental references

1. Taher AT, et al. Thalassaemia. *Lancet.* 2018;391(10116):155-167.

2. Yuan J, et al. Isolation, characterization, and immunoprecipitation studies of immune complexes from membranes of beta-thalassemic erythrocytes. *Blood.* 1992;79(11):3007-3013.

3. Punjani A, et al. cryoSPARC: algorithms for rapid unsupervised cryo-EM structure determination. *Nat Methods.* 2017;14(3):290-296.

4. Zivanov J, et al. New tools for automated high-resolution cryo-EM structure determination in RELION-3. *Elife.* 2018;7.

5. Zhang K. Gctf: Real-time CTF determination and correction. *J Struct Biol.* 2016;193(1):1-12.

6. Emsley P, et al. Features and development of Coot. *Acta Crystallogr D Biol Crystallogr.* 2010;66(Pt 4):486-501.

7. Afonine PV, et al. Real-space refinement in PHENIX for cryo-EM and crystallography. *Acta Crystallogr D Struct Biol.* 2018;74(Pt 6):531-544.

8. Jumper J, et al. Highly accurate protein structure prediction with AlphaFold. *Nature.* 2021;596(7873):583-589.

9. Varadi M, et al. AlphaFold Protein Structure Database in 2024: providing structure coverage for over 214 million protein sequences. *Nucleic Acids Res.* 2024;52(D1):D368-D375.

10. Prisant MG, et al. New tools in MolProbity validation: CaBLAM for CryoEM backbone, UnDowser to rethink "waters," and NGL Viewer to recapture online 3D graphics. *Protein Sci.* 2020;29(1):315-329.

11. Schroedinger L. 2010.

12. Pettersen EF, et al. UCSF ChimeraX: Structure visualization for researchers, educators, and developers. *Protein Sci.* 2021;30(1):70-82.

13. Pettersen EF, et al. UCSF Chimera--a visualization system for exploratory research and analysis. *J Comput Chem.* 2004;25(13):1605-1612.

14. Rivella S. beta-thalassemias: paradigmatic diseases for scientific discoveries and development of innovative therapies. *Haematologica.* 2015;100(4):418-430.

15. Rivella S. The role of ineffective erythropoiesis in non-transfusion-dependent thalassemia. *Blood Rev.* 2012;26(Suppl 1):S12-15.

16. Nemeth E, et al. Hepcidin regulates cellular iron efflux by binding to ferroportin and inducing its internalization. *Science.* 2004;306(5704):2090-2093.

17. Nemeth E. Hepcidin biology and therapeutic applications. *Expert Rev Hematol.* 2014;3(2):153-155.

18. Park CH, et al. Hepcidin, a urinary antimicrobial peptide synthesized in the liver. *J Biol Chem.* 2001;276(11):7806-7810.

Supplemental Table 1. Inclusion and exclusion criteria

Inclusion Criteria	Exclusion Criteria
Male or female 18–60 years of age at the screening visit	Members of the clinical site study team and/or their immediate family, unless prior approval granted by the sponsor.
BMI between 18–32 kg/m^2,	Pregnant or breastfeeding female participants<br>Consistent with Clinical Trial Facilitation Group guidance, female participants of childbearing potential (WOCBP)* who are unwilling to practice highly effective contraception during the study through the end-of-study visit. Highly effective contraceptive measures include:<br>a) Stable use of combined (estrogen- and progestogen- containing) hormonal contraception (oral, intravaginal, transdermal) or progestogen-only hormonal contraception (oral, injectable, implantable) associated with inhibition of ovulation initiated 2 or more menstrual cycles prior to screening<br>b) IUD; intra-uterine hormone-releasing system<br>c) Bilateral tubal ligation or tubal occlusion<br>d) Vasectomized partner (provided that the male vasectomized partner is the sole sexual partner of the female study participant and that the vasectomized partner has obtained medical assessment of surgical success for the procedure); and/or<br>e) Sexual abstinence[†]
Judged by the investigator to be in good health based on medical history, physical examination, vital sign measurements, and ECGs performed at screening and/or prior to administration of the initial dose of the study drug	In addition, premenopausal female participants whose method(s) of birth control is/are associated with ongoing menstruation (eg, combined hormonal contraceptive regimens associated with withdrawal bleeding, non-hormone-releasing IUD, bilateral tubal ligation, bilateral salpingectomy, vasectomized partner, sexual abstinence). Female participants must not be menstruating during the trial, due to being postmenopausal[,‡] or due to permanent sterilization via hysterectomy, and/or bilateral oophorectomy, or amenorrheic due to use of hormone-releasing IUD, implantable device, or intake of continuous hormonal contraception.
In good health based on laboratory safety testing obtained at the screening and baseline visits<br>Hemoglobin, serum iron, transferrin, serum ferritin, and transferrin saturation, equal to or above the lower limit of the reference range for the participant's age and sex at the local labs, at screening, repeatable once during the screening period	Sexually active male participants with female partners who are unwilling to use the following forms of medically acceptable birth control during the study through the EOS end-of-study visit: vasectomy with medical assessment of surgical success OR consistent use of a condom.
WBC count, platelet count, RBC count, hematocrit, and RBC hemoglobin not clinically significantly outside of the reference range in the	History of clinically significant cardiovascular (including congestive heart failure and angina), respiratory, hepatic, renal, gastrointestinal, endocrine, hematologic, infectious,

judgment of the investigator at the screening and baseline visits

Willing and able to comply with clinic visits and study-related procedures

Provides signed informed consent

autoimmune, oncologic, psychiatric, or neurologic disease, as assessed by the investigator, that may confound the results of the study or poses an additional risk to the participant by study participation.

History of chronic anemia

History of RBC transfusion reaction

Presents any concern to the study investigator that might confound the results of the study or poses an additional risk to the participant by their participation in the study

Was hospitalized (ie, >24 h) for any reason within 30 days of the screening visit

Whole blood donation within the previous 56 days or plasma donation within the previous 7 days prior to screening. Planning on whole blood or plasma donation at any time point during the study.

Has an estimated glomerular filtration rate of <60 mL/min/1.73m^2 at the screening visit

Is a current smoker or former smoker, including e-cigarettes, who stopped smoking within 3 months prior to the screening visit

Has a confirmed positive drug or alcohol test result at the screening visit and/or prior to randomization; or a history of recreational drug use (eg, marijuana) and/or drug or alcohol abuse within a year prior to the screening visit

History of recurrent syncopal episodes

History of symptomatic orthostatic hypotension

Positive for HIV or HBsAg at the screening visit

Positive for hepatitis C Ab at the screening visit

Any malignancy within the past 10 years (except for non-melanoma skin cancer or cervical/anus in-situ cancer, that have been resected with no evidence of metastatic disease for 3 years prior to the screening visit)

Has a history of significant multiple and/or severe allergies (eg, latex gloves), or has had an anaphylactic reaction to prescription or nonprescription drugs or food

Participated in any clinical research study evaluating another investigational drug including biologics or therapy, including specific immunotherapy, within 90 days or at least 5 half-lives (whichever is longer) of an investigational biologic drug, or at least 4 weeks for other investigational drug, prior to the screening visit

Use of any prescription and nonprescription medications or nutritional supplements from approximately 2 weeks or 5

	half-lives, whichever is longer, prior to first administration of the study drug through the end of the study, except contraceptives as noted above, and the permitted medications listed<br><br>Has received a COVID-19 vaccination within 1 week of planned start of study medication or for which the planned COVID-19 vaccinations would not be completed 1 week prior to the start of the study drug<br>Planned elective surgery during the trial

FSH, follicle-stimulating hormone; IUD, intra-uterine device.

*Female participants of childbearing potential are defined as people of female sex who are fertile following menarche until becoming postmenopausal, unless permanently sterile. Permanent sterilization methods include hysterectomy, bilateral salpingectomy, and bilateral oophorectomy.

†Sexual abstinence is considered a highly effective method only if defined as refraining from heterosexual intercourse during the entire period of risk associated with the study drugs. The reliability of sexual abstinence needs to be evaluated in relation to the duration of the clinical trial and the preferred and usual lifestyle of the participant. Periodic abstinence (calendar, symptothermal, post-ovulation methods), withdrawal (coitus interruptus), spermicides only, and lactational amenorrhea method are not acceptable methods of contraception. Female condom and male condom should not be used together.

‡A postmenopausal state is defined as no menses for 12 months without an alternative medical cause. A high follicle FSH level in the postmenopausal range may be used to confirm a postmenopausal state in female participants not using hormonal contraception or hormonal replacement therapy. However, in the absence of 12 months of amenorrhea, 2 separate FSH measurements are required to determine the existence of a postmenopausal state

Supplemental Table 2. Overview of treatment-emergent adverse events for the i.v. cohort

**Participants with at least 1, n (%)**	**Pooled placebo i.v. (*n* = 10)**	**REGN7999 10 mg i.v. (*n* = 6)**	**REGN7999 30 mg i.v. (*n* = 6)**	**REGN7999 100 mg i.v. (*n* = 6)**	**REGN7999 300 mg i.v. (*n* = 6)**	**REGN7999 900 mg i.v. (*n* = 6)**	**Total REGN7999 IV (*n* = 30)**
TEAE	10 (100)	5 (83.3)	6 (100)	6 (100)	5 (83.3)	5 (83.3)	27 (90.0)
Severe TEAE	1 (10.0)	0	0	1 (16.7)	0	0	1 (3.3)
Treatment-emergent serious adverse event	0	0	0	0	0	0	0
Treatment-related TEAE	3 (30.0)	2 (33.3)	2 (33.3)	5 (83.3)	1 (16.7)	2 (33.3)	12 (40.0)
TEAE leading to drug interruption	0	0	0	0	0	0	0
TEAE leading to study discontinuation	0	0	0	0	0	0	0
TEAE leading to death	0	0	0	0	0	0	0

IV = intravenous; TEAE= treatment-emergent adverse event; TE-SAE= treatment-emergent serious adverse event

MedDRA (Version 26.0) coding dictionary applied

The end of study (EOS) visit occurs on week 20 for IV cohorts 1 to 4 and for SC cohorts 1 to 2. For IV cohort 5 and SC cohort 3, the EOS visit occurs on week 26.

Supplemental Table 3. Overview of treatment-emergent adverse events for the s.c. cohort

Participants with at least 1, n (%)	Pooled placebo s.c. (*n* = 6)	REGN7999 100 mg s.c. (*n* = 6)	REGN7999 300 mg s.c. (*n* = 6)	REGN7999 900 mg s.c. (*n* = 6)	Total REGN7999 s.c. (*n* = 18)
TEAE	4 (66.7)	6 (100)	5 (83.3)	6 (100)	17 (94.4)
Severe TEAE	0	0	0	0	0
Treatment-emergent serious adverse event	0	0	0	0	0
Treatment-related TEAE	2 (33.3)	4 (66.7)	1 (16.7)	3 (50.0)	8 (44.4)
TEAE leading to study discontinuation	0	0	0	0	0
TEAE leading to death	0	0	0	0	0

TEAE, treatment-emergent adverse event.

Medical Dictionary for Regulatory Activities (version 26.0) coding was applied.

The end-of-study visit occurred in week 20 for i.v. cohorts 1 to 4 and for s.c. cohorts 1 to 2. For i.v. cohort 5 and s.c. cohort 3, the end-of-study visit occurred in week 26.

Supplemental Table 4. Overview of TEAEs by system organ class and preferred term (i.v. cohort)

n (%)	Pooled placebo i.v. (*n* = 10)	REGN7999 10 mg i.v. (*n* = 6)	REGN7999 30 mg i.v. (*n* = 6)	REGN7999 100 mg i.v. (*n* = 6)	REGN7999 300 mg i.v. (*n* = 6)	REGN7999 900 mg i.v. (*n* = 6)	Total REGN7999 i.v. (*n* = 30)
Number of participants with at least 1 TEAE	10 (100)	5 (83.3)	6 (100)	6 (100)	5 (83.3)	5 (83.3)	27 (90.0)
Nervous system disorders	7 (70.0)	4 (66.7)	3 (50.0)	5 (83.3)	2 (33.3)	2 (33.3)	16 (53.3)
Headache	7 (70.0)	4 (66.7)	1 (16.7)	3 (50.0)	2 (33.3)	2 (33.3)	12 (40.0)
Dizziness	0	1 (16.7)	0	2 (33.3)	0	0	3 (10.0)
Paresthesia	0	0	0	1 (16.7)	0	1 (16.7)	2 (6.7)
Dizziness postural	0	0	1 (16.7)	0	0	0	1 (3.3)
Migraine	1 (10.0)	0	0	1 (16.7)	0	0	1 (3.3)
Presyncope	0	0	0	0	1 (16.7)	0	1 (3.3)
Syncope	0	0	1 (16.7)	0	0	0	1 (3.3)
Infections and infestations	4 (40.0)	2 (33.3)	4 (66.7)	4 (66.7)	2 (33.3)	2 (33.3)	14 (46.7)
Nasopharyngitis	2 (20.0)	2 (33.3)	3 (50.0)	2 (33.3)	0	1 (16.7)	8 (26.7)
Gastroenteritis	0	0	2 (33.3)	1 (16.7)	0	0	3 (10.0)
Influenza	0	0	2 (33.3)	0	0	0	2 (6.7)
Upper respiratory tract infection	0	0	0	2 (33.3)	0	0	2 (6.7)

Cystitis	0	0	0	0	1 (16.7)	0	1 (3.3)
Cytomegalovirus infection	0	0	0	0	0	1 (16.7)	1 (3.3)
Erysipelas	0	0	1 (16.7)	0	0	0	1 (3.3)
Otitis externa	0	0	0	0	0	1 (16.7)	1 (3.3)
Urethritis	0	0	0	0	1 (16.7)	0	1 (3.3)
Pharyngitis	1 (10.0)	0	0	0	0	0	0
Urinary tract infection	1 (10.0)	0	0	0	0	0	0
Respiratory, thoracic, and mediastinal disorders	4 (40.0)	5 (83.3)	2 (33.3)	4 (66.7)	1 (16.7)	2 (33.3)	14 (46.7)
Oropharyngeal pain	2 (20.0)	3 (50.0)	2 (33.3)	2 (33.3)	0	1 (16.7)	8 (26.7)
Cough	0	1 (16.7)	0	0	0	2 (33.3)	3 (10.0)
Rhinorrhea	0	0	0	2 (33.3)	0	1 (16.7)	3 (10.0)
Nasal congestion	2 (20.0)	1 (16.7)	0	0	1 (16.7)	0	2 (6.7)
Dysphonia	1 (10.0)	0	0	1 (16.7)	0	0	1 (3.3)
Hyperventilation	0	1 (16.7)	0	0	0	0	1 (3.3)
Sneezing	0	1 (16.7)	0	0	0	0	1 (3.3)
Gastrointestinal disorders	3 (30.0)	1 (16.7)	3 (50.0)	3 (50.0)	2 (33.3)	1 (16.7)	10 (33.3)
Nausea	1 (10.0)	0	2 (33.3)	1 (16.7)	1 (16.7)	0	4 (13.3)
Diarrhea	0	0	0	1 (16.7)	1 (16.7)	1 (16.7)	3 (10.0)

Toothache	1 (10.0)	1 (16.7)	0	1 (16.7)	1 (16.7)	0	3 (10.0)
Abdominal pain	2 (20.0)	0	0	1 (16.7)	0	1 (16.7)	2 (6.7)
Vomiting	1 (10.0)	0	1 (16.7)	0	1 (16.7)	0	2 (6.7)
Gastroesophageal reflux disease	0	0	1 (16.7)	0	0	0	1 (3.3)
General disorders and administration-site conditions	1 (10.0)	2 (33.3)	1 (16.7)	3 (50.0)	1 (16.7)	3 (50.0)	10 (33.3)
Fatigue	0	1 (16.7)	0	2 (33.3)	0	2 (33.3)	5 (16.7)
Puncture-site hematoma	0	1 (16.7)	0	0	1 (16.7)	0	2 (6.7)
Chills	0	0	0	1 (16.7)	0	0	1 (3.3)
Influenza-like illness	0	0	1 (16.7)	0	0	0	1 (3.3)
Non-cardiac chest pain	0	0	0	0	0	1 (16.7)	1 (3.3)
Puncture site pain	0	0	0	0	1 (16.7)	0	1 (3.3)
Catheter site pain	1 (10.0)	0	0	0	0	0	0
Investigations	1 (10.0)	1 (16.7)	2 (33.3)	0	2 (33.3)	1 (16.7)	6 (20.0)
Blood creatine phosphokinase increased	0	0	1 (16.7)	0	0	1 (16.7)	2 (6.7)
Blood potassium increased	1 (10.0)	0	0	0	1 (16.7)	0	1 (3.3)
Eosinophil count increased	0	1 (16.7)	0	0	0	0	1 (3.3)

Lymphocyte count increased	0	0	1 (16.7)	0	0	0	1 (3.3)
WBC count decreased	0	0	0	0	1 (16.7)	0	1 (3.3)
Musculoskeletal and connective tissue disorders	3 (30.0)	2 (33.3)	0	1 (16.7)	0	2 (33.3)	5 (16.7)
Arthralgia	0	2 (33.3)	0	0	0	1 (16.7)	3 (10.0)
Pain in extremity	0	0	0	1 (16.7)	0	1 (16.7)	2 (6.7)
Flank pain	0	0	0	1 (16.7)	0	0	1 (3.3)
Myalgia	0	1 (16.7)	0	0	0	0	1 (3.3)
Neck pain	1 (10.0)	1 (16.7)	0	0	0	0	1 (3.3)
Musculoskeletal chest pain	1 (10.0)	0	0	0	0	0	0
Musculoskeletal stiffness	1 (10.0)	0	0	0	0	0	0
Skin and subcutaneous tissue disorders	1 (10.0)	1 (16.7)	1 (16.7)	1 (16.7)	1 (16.7)	1 (16.7)	5 (16.7)
Dermatitis contact	1 (10.0)	1 (16.7)	0	0	0	1 (16.7)	2 (6.7)
Pityriasis rosea	0	0	1 (16.7)	0	0	0	1 (3.3)
Pruritus	0	0	0	1 (16.7)	0	0	1 (3.3)
Rosacea	0	0	0	0	1 (16.7)	0	1 (3.3)
Injury, poisoning, and procedural complications	0	0	1 (16.7)	0	1 (16.7)	0	2 (6.7)

Animal bite	0	0	0	0	1 (16.7)	0	1 (3.3)
Thermal burn	0	0	1 (16.7)	0	0	0	1 (3.3)
Ear and labyrinth disorders	0	0	0	0	0	1 (16.7)	1 (3.3)
Cerumen impaction	0	0	0	0	0	1 (16.7)	1 (3.3)
Psychiatric disorders	1 (10.0)	0	0	0	0	1 (16.7)	1 (3.3)
Insomnia	0	0	0	0	0	1 (16.7)	1 (3.3)
Nervousness	1 (10.0)	0	0	0	0	0	0
Renal and urinary disorders	0	0	0	1 (16.7)	0	0	1 (3.3)
Ureterolithiasis	0	0	0	1 (16.7)	0	0	1 (3.3)

TEAE, treatment-emergent adverse event.

Medical Dictionary for Regulatory Activities (version 26.0) coding was applied.

A participant who reported 2 or more TEAEs with the same preferred term was counted only once for that term.

A participant who reported 2 or more TEAEs with different preferred terms within the same system organ class was counted only once in that system organ class.

System organ class and preferred term are sorted by decreasing frequency of the total group.

The end-of-study visit occurred in week 20 for i.v. cohorts 1 to 4 . For i.v. cohort 5 , the end-of-study visit occurred in week 26.

Supplemental Table 5. Overview of TEAEs by system organ class and preferred term (s.c. cohort)

n (%)	Pooled placebo s.c. (*n* = 6)	REGN7999 100 mg s.c. (*n* = 6)	REGN7999 300 mg s.c. (*n* = 6)	REGN7999 900 mg s.c. (*n* = 6)	Total REGN7999 s.c. (*n* = 18)
Number of participants with at least 1 TEAE	4 (66.7)	6 (100)	5 (83.3)	6 (100)	17 (94.4)
Infections and infestations	1 (16.7)	2 (33.3)	3 (50.0)	4 (66.7)	9 (50.0)
Nasopharyngitis	1 (16.7)	1 (16.7)	0	4 (66.7)	5 (27.8)
Upper respiratory tract infection	0	1 (16.7)	1 (16.7)	0	2 (11.1)
Oral herpes	1 (16.7)	0	1 (16.7)	0	1 (5.6)
Pharyngitis	0	0	1 (16.7)	0	1 (5.6)
Skin infection	0	0	0	1 (16.7)	1 (5.6)
Nervous system disorders	1 (16.7)	4 (66.7)	3 (50.0)	2 (33.3)	9 (50.0)
Headache	1 (16.7)	4 (66.7)	2 (33.3)	2 (33.3)	8 (44.4)
Dizziness	1 (16.7)	0	1 (16.7)	1 (16.7)	2 (11.1)
Migraine	0	0	1 (16.7)	0	1 (5.6)
General disorders and administration-site conditions	3 (50.0)	3 (50.0)	0	5 (83.3)	8 (44.4)
Fatigue	3 (50.0)	1 (16.7)	0	2 (33.3)	3 (16.7)
Injection-site reaction	1 (16.7)	0	0	3 (50.0)	3 (16.7)

Generalized edema	0	1 (16.7)	0	0	1 (5.6)
Malaise	0	1 (16.7)	0	0	1 (5.6)
Pain	0	0	0	1 (16.7)	1 (5.6)
Puncture-site hematoma	1 (16.7)	0	0	1 (16.7)	1 (5.6)
Puncture-site pain	1 (16.7)	0	0	0	0
Gastrointestinal disorders	1 (16.7)	2 (33.3)	1 (16.7)	4 (66.7)	7 (38.9)
Diarrhea	0	0	0	3 (50.0)	3 (16.7)
Nausea	0	0	1 (16.7)	2 (33.3)	3 (16.7)
Abdominal pain	0	0	1 (16.7)	1 (16.7)	2 (11.1)
Dyspepsia	0	1 (16.7)	0	0	1 (5.6)
Toothache	1 (16.7)	1 (16.7)	0	0	1 (5.6)
Vomiting	0	0	0	1 (16.7)	1 (5.6)
Investigations	0	3 (50.0)	1 (16.7)	0	4 (22.2)
Blood creatine phosphokinase increased	0	2 (33.3)	1 (16.7)	0	3 (16.7)
WBC count decreased	0	1 (16.7)	0	0	1 (5.6)
Respiratory, thoracic, and mediastinal disorders	3 (50.0)	0	1 (16.7)	3 (50.0)	4 (22.2)
Oropharyngeal pain	3 (50.0)	0	1 (16.7)	1 (16.7)	2 (11.1)

Rhinorrhea	0	0	0	2 (33.3)	2 (11.1)
Dry throat	0	0	0	1 (16.7)	1 (5.6)
Throat irritation	0	0	0	1 (16.7)	1 (5.6)
Injury, poisoning, and procedural complications	1 (16.7)	2 (33.3)	0	1 (16.7)	3 (16.7)
Arthropod bite	1 (16.7)	0	0	1 (16.7)	1 (5.6)
Epicondylitis	0	1 (16.7)	0	0	1 (5.6)
Wound	0	1 (16.7)	0	0	1 (5.6)
Musculoskeletal and connective tissue disorders	1 (16.7)	1 (16.7)	0	2 (33.3)	3 (16.7)
Back pain	0	0	0	1 (16.7)	1 (5.6)
Myalgia	0	0	0	1 (16.7)	1 (5.6)
Rotator cuff syndrome	0	1 (16.7)	0	0	1 (5.6)
Pain in extremity	1 (16.7)	0	0	0	0
Cardiac disorders	0	0	1 (16.7)	0	1 (5.6)
Palpitations	0	0	1 (16.7)	0	1 (5.6)
Ear and labyrinth disorders	0	0	1 (16.7)	0	1 (5.6)
Tinnitus	0	0	1 (16.7)	0	1 (5.6)
Psychiatric disorders	1 (16.7)	0	0	1 (16.7)	1 (5.6)
Nervousness	0	0	0	1 (16.7)	1 (5.6)

Insomnia	1 (16.7)	0	0	0	0
Reproductive system and breast disorders	0	1 (16.7)	0	0	1 (5.6)
Gynecomastia	0	1 (16.7)	0	0	1 (5.6)
Skin and subcutaneous tissue disorders	1 (16.7)	0	0	1 (16.7)	1 (5.6)
Dry skin	1 (16.7)	0	0	1 (16.7)	1 (5.6)

Medical Dictionary for Regulatory Activities (version 26.0) coding was applied.

A participant who reported 2 or more TEAEs with the same preferred term was counted only once for that term.

A participant who reported 2 or more TEAEs with different preferred terms within the same system organ class was counted only once in that system organ class.

System organ class and preferred term are sorted by decreasing frequency of the total group.

The end-of-study visit occurred in week 20 for s.c. cohorts 1 to 2. For s.c. cohort 3, the end=of-study visit occurred in week 26.

Supplemental Table 6. CryoEM data collection, map calculation, and structure refinement

	TMPRSS6/REGN7999 Fab/REGN8023 Fab complex
**Data collection and processing**	
Magnification	105,000
Voltage (kV)	300
Electron exposure (e–/Å2)	~40
Defocus range (μm)	-1.4 to -2.4
Pixel size (Å)	0.85
Number of movies collected	3,580
Initial number of particles	1,455,534
Particles selected after 2D classification	1,034,494
Final selected particles	105,142
Symmetry imposed	C1
Map resolution (Å)	3.29
FSC threshold	0.143
**Model Refinement and validation**	
Map sharpening B factor (Å2)	-76
Initial model	AlphaFold (TMPRSS6)<br>Homology model (Fabs)
Model composition	
Non-hydrogen atoms	11,971
Protein residues	1,587
Ligands	12 (CA:3, NAG:9)
R.m.s. deviations	
Bond lengths (Å)	0.003

Bond angles (°)	0.730
Validation	
MolProbity score	1.83
Rotamer outliers (%)	0.08
Clash score	8.97
Ramachandran plot	
Favored (%)	94.90
Allowed (%)	4.97
Disallowed (%)	0.13
**Deposition ID**	
PDB	9NRC
EMDB	49728

Supplemental Figure 1. REGN7999 blocked human and mouse TMPRSS6 activity and prevents HJV cleavage in vitro. Soluble HJV was measured by ELISA in HEK293 cells that overexpress (A) human or (B) mouse TMPRSS6. (C, D) Extracellular HJV was measured using flow cytometry in the same cell lines

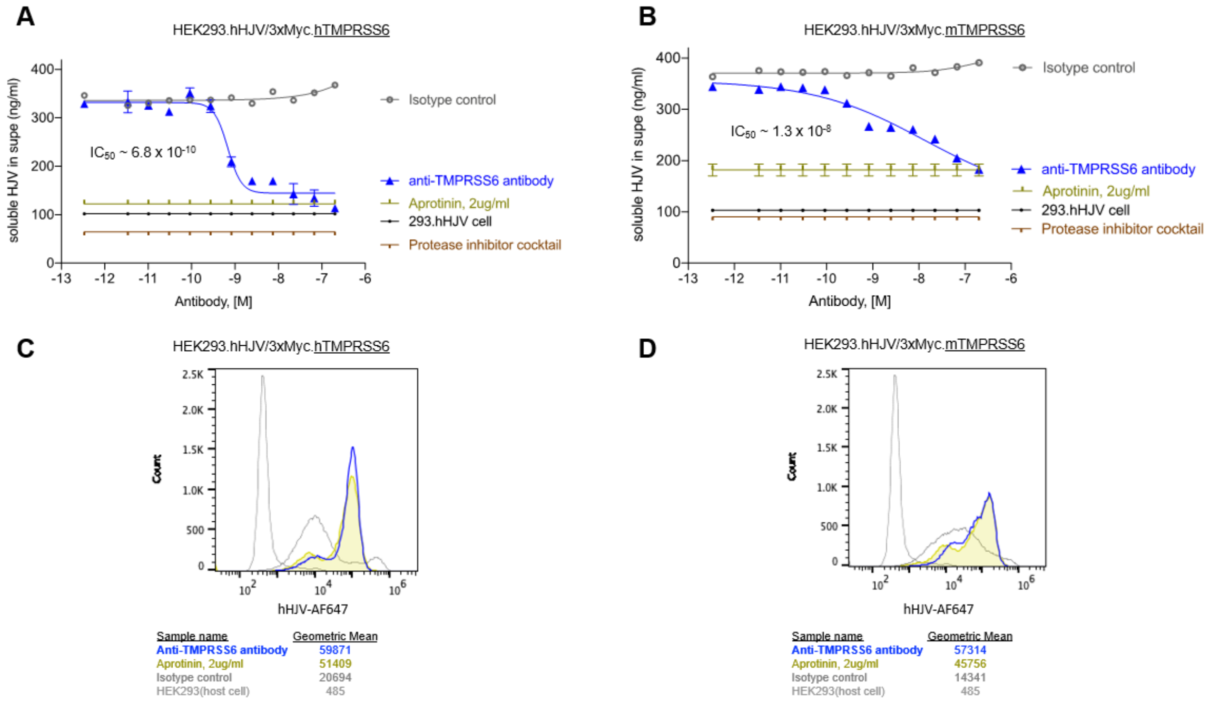

HJV, hemojuvelin.

In all experiments cells were treated with REGN7999, a protease inhibitor cocktail, or aprotinin at different concentrations.

Supplemental Figure 2. REGN7999 bound TMPRSS6 across species. Binding of REGN7999 to different variants of human, mouse, and monkey TMPRSS6 expressed on HEK293 cells was evaluated using flow cytometry with EC_{50} values listed in the figures. REGN7999 binding to (A–D) human reference and variant TMPRSS6, (E) macaca fascicularis (Cynomolgus monkey) TMPRSS6, and (F) mouse TMPRSS6. (G) REGN7999 showed no binding to parental HEK293 cells.

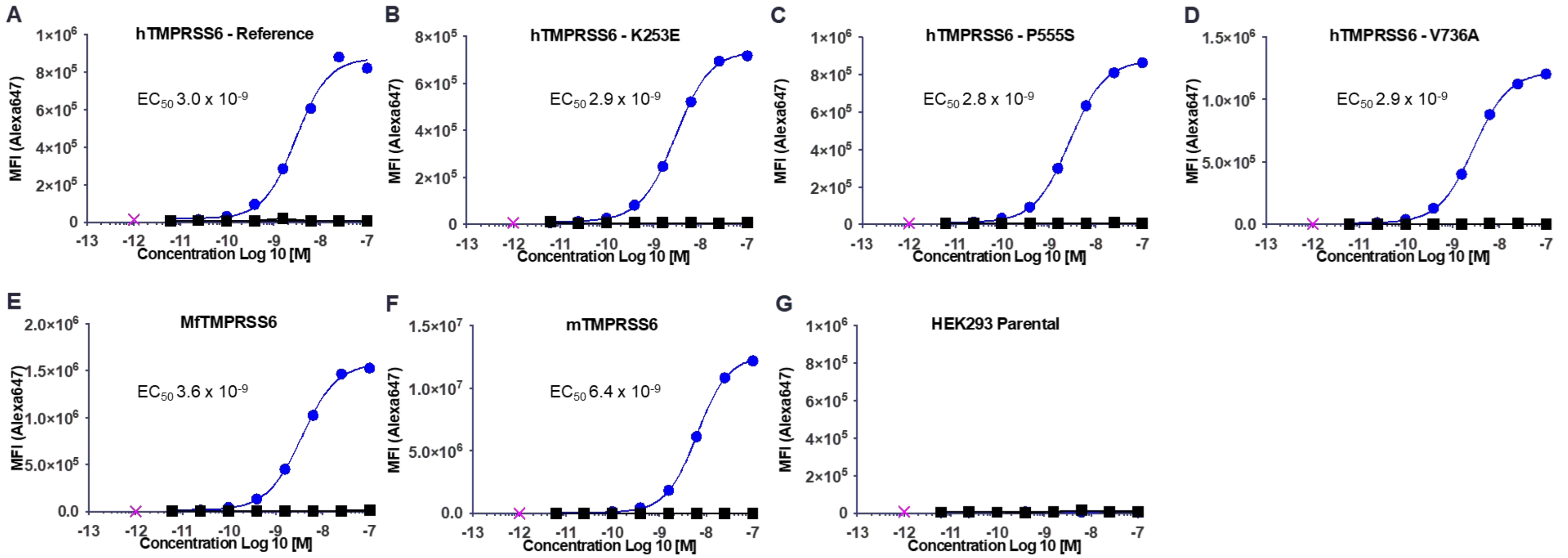

Supplemental Figure 3. REGN7999 inhibited TMPRSS6 activity across species and independent of variant. Substrate cleavage assays showed REGN7999 reduced proteolytic activity of (A–D) human reference and variant TMPRSS6, (E) cynomolgus monkey TMPRSS6, and (F) mouse TMPRSS6.

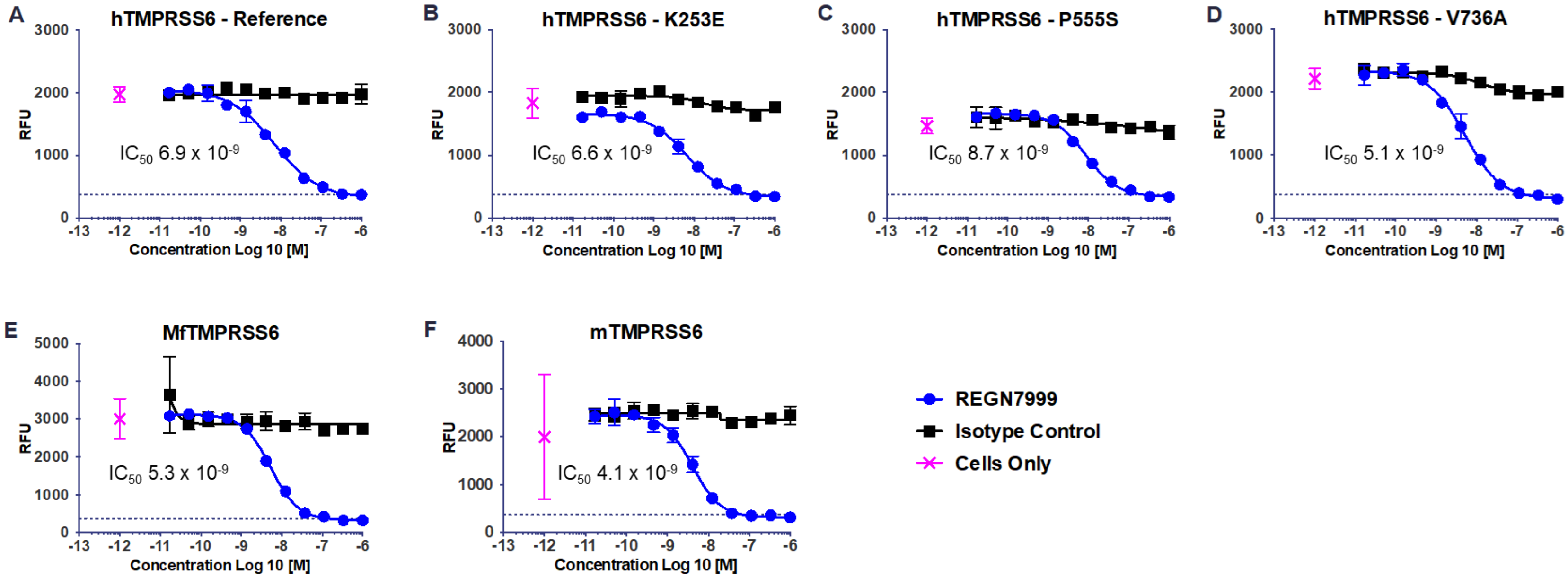

RFU, relative fluorescence unit.

Supplemental Figure 4. CryoEM structure of TMPRSS6/REGN7999 complex. **Exemplary 2D class averages (A), Fourier shell correlation (FSC) curve of final map with solvent mask applied (B), and map colored according to local resolution (C) of TMPRSS6/REGN7999 Fab/REGN8023 Fab complex. (D) Domain architecture of TMPRSS6. (E) CryoEM map with TMPRSS6 colored by domain as in (D) and REGN7999 heavy and light chains colored forest green and light green, respectively. REGN8023 Fab (gray/white) was added to the complex as a fiducial mark to facilitate cryoEM structure determination. (F) Surface representation of TMPRSS6 structure, colored as in (E) and (F). Note that the REGN7999 epitope (colored forest green, 4 Å distance cutoff) does not cover the catalytic triad (magenta). Details of REGN7999 epitope and paratope at the catalytic domain (G) and LDLRA2 domain (H). Epitope and paratope residues (4 Å interatomic distance cutoff) are shown as stick and labeled.**

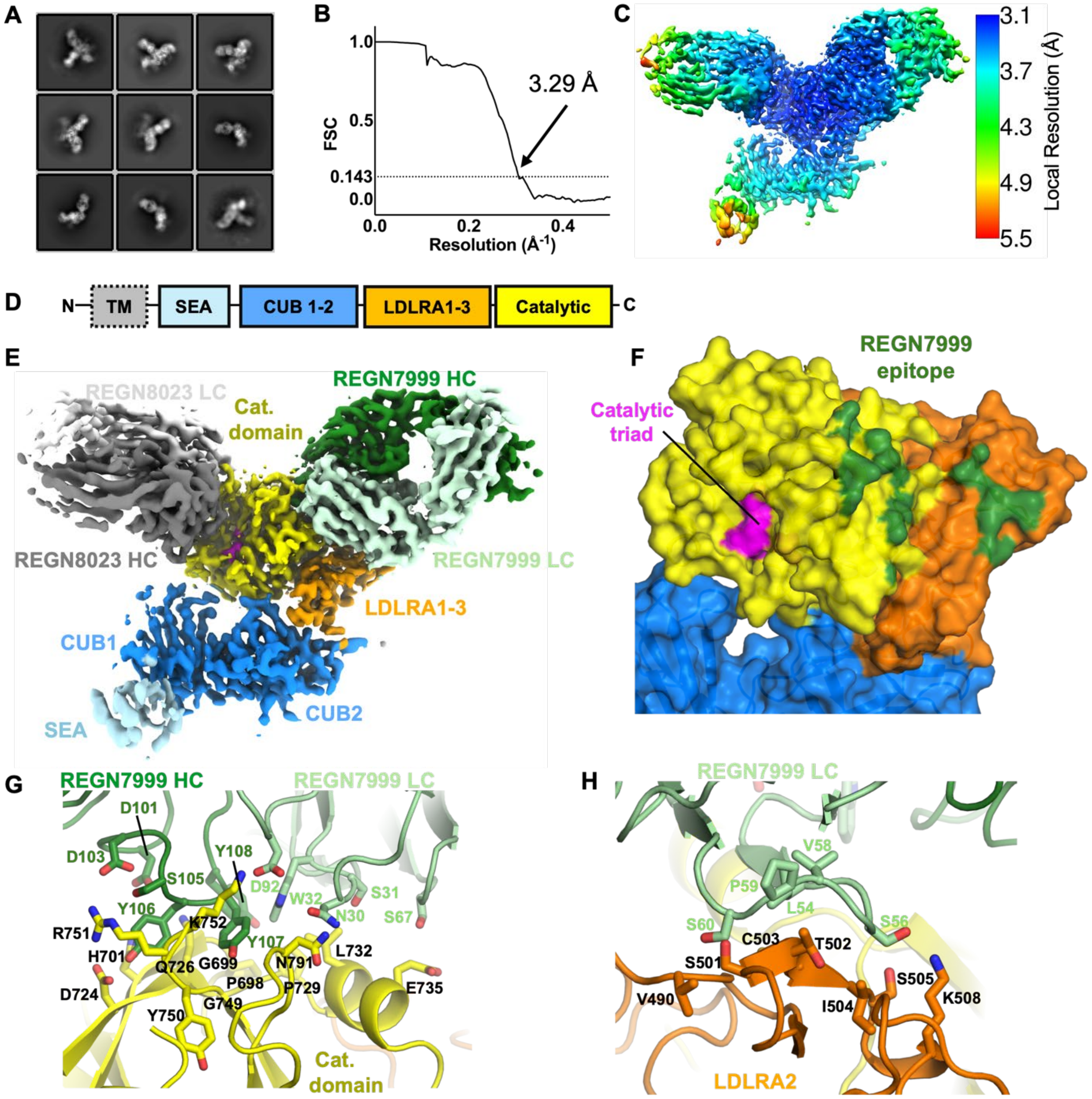
A
B
1.0
0.5
0.143
0.0
FSC
3.29 Å
0.0
0.2
0.4
Resolution (Å-1)
C
3.1
3.7
4.3
4.9
5.5
Local Resolution (Å)
D
N
TM
SEA
CUB 1-2
LDLRA1-3
Catalytic
C
E
REGN8023 LC
Cat. domain
REGN7999 HC
REGN8023 HC
REGN7999 LC
LDLRA1-3
CUB1
CUB2
SEA
F
REGN7999 epitope
Catalytic triad
G
REGN7999 HC
REGN7999 LC
D101
D103
S105
Y108
D92
W32
S31
N30
S67
Y106
K752
Y107
R751
H701
Q726
G699
N791
L732
P698
P729
E735
D724
G749
Y750
Cat. domain
H
REGN7999 LC
V58
P59
L54
S60
S56
C503
T502
S501
S505
V490
I504
K508
LDLRA2

Supplemental Figure 5. REGN7999 reduced serum iron in Cynomolgus monkey for 6 weeks and underwent target-mediated clearance. All animals received 1 bolus injection of REGN7999 (15 mg/kg) or isotype control. (A) Serum iron levels and (B) serum hepcidin levels during the 8 weeks of the experiment.

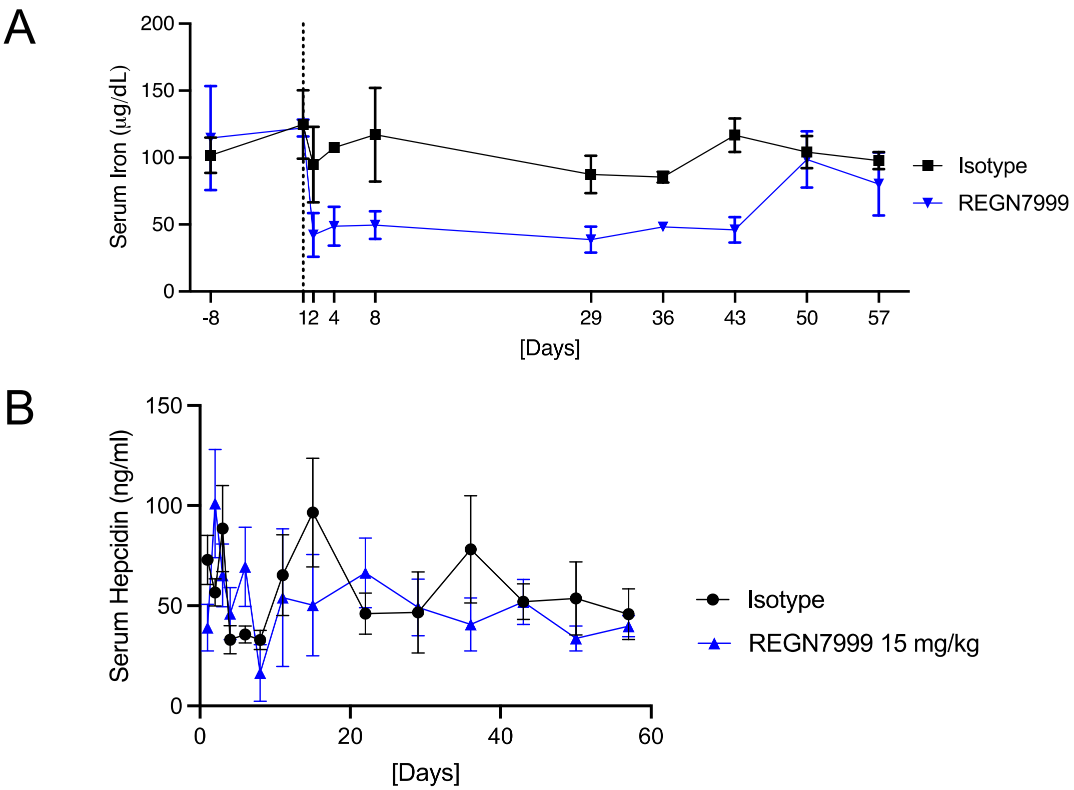

NHP, non-human primate.

Supplemental Figure 6. Body and liver weight remained similar between different treatment groups. (A) Body weight of WT littermates and Hbb$^{th3/+}$ mice (injected with REGN7999 or isotype control) was measured weekly until termination of the study. (B) Liver weights of WT littermates and Hbb$^{th3/+}$ mice (injected with REGN7999 or isotype control) at termination of the experiment.

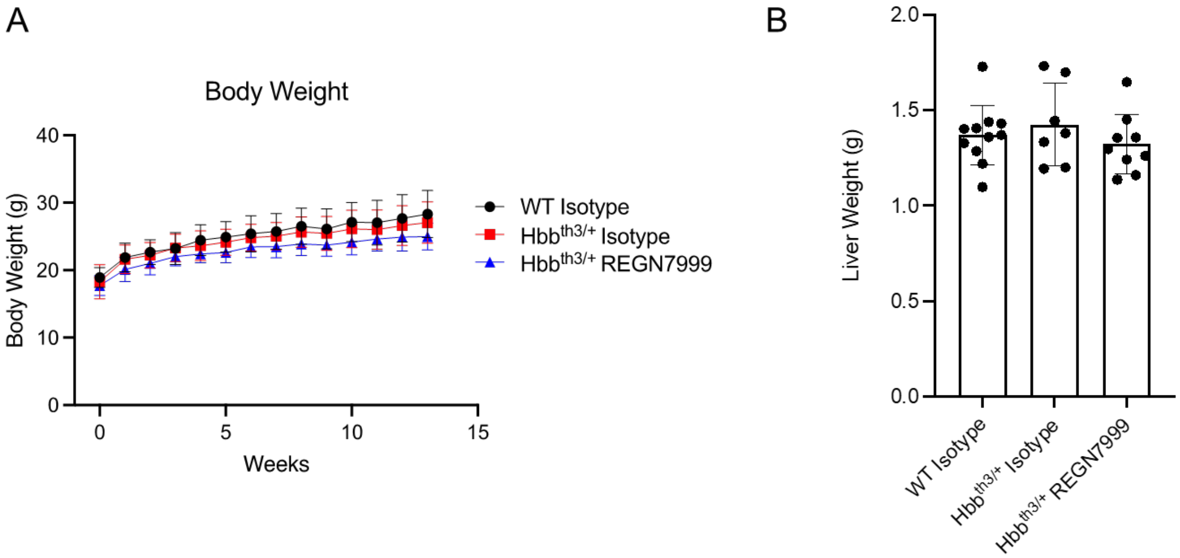

Statistics were calculated with 1-way ANOVA. A statistically significant difference was reached at $P < 0.05$.

Supplemental Figure 7. REGN7999 treatment did not lead to liver damage in $Hbb^{th3/+}$ mice. Serum levels of (A) AST and (B) ALT were measured after 8 weeks of injections of isotype control or REGN7999.

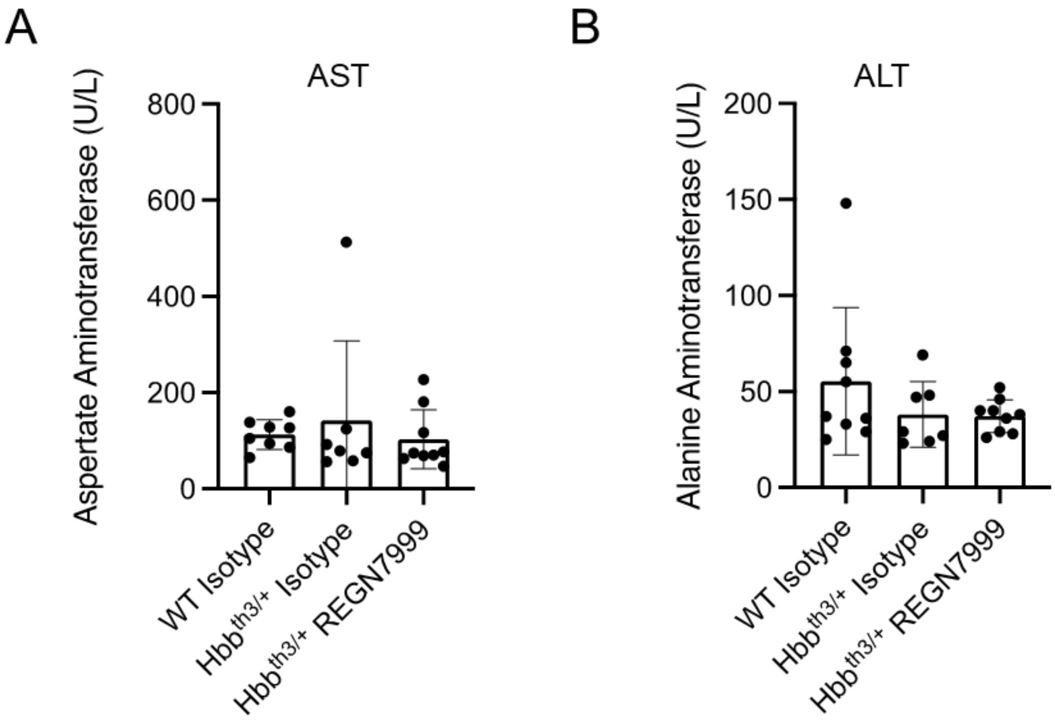

ALT, alanine aminotransferase; AST, aspartate aminotransferase.

Supplemental Figure 8. REGN7999 treatment did not reduce splenic iron loading. (A) Representative images of Perl's stain on spleens of Hbb$^{th3/+}$ mice or WT littermates injected with isotype control or REGN7999. Upper row are images at 2x magnification, lower row at 40x magnification; the blue color indicates iron accumulation. (B) Bar graph depicturing the percentage of the area stained by Perl's stain. (C) Bar graph of total spleen iron. (D) Bar graph of spleen weight. (E) Representative images of Perl's stain on duodenum cross-sections of Hbb$^{th3/+}$ mice or WT littermates injected with isotype control or REGN7999. Upper row are images at 4x magnification, lower row at 40x magnification. (F) Bar graph of the analysis results from the staining.

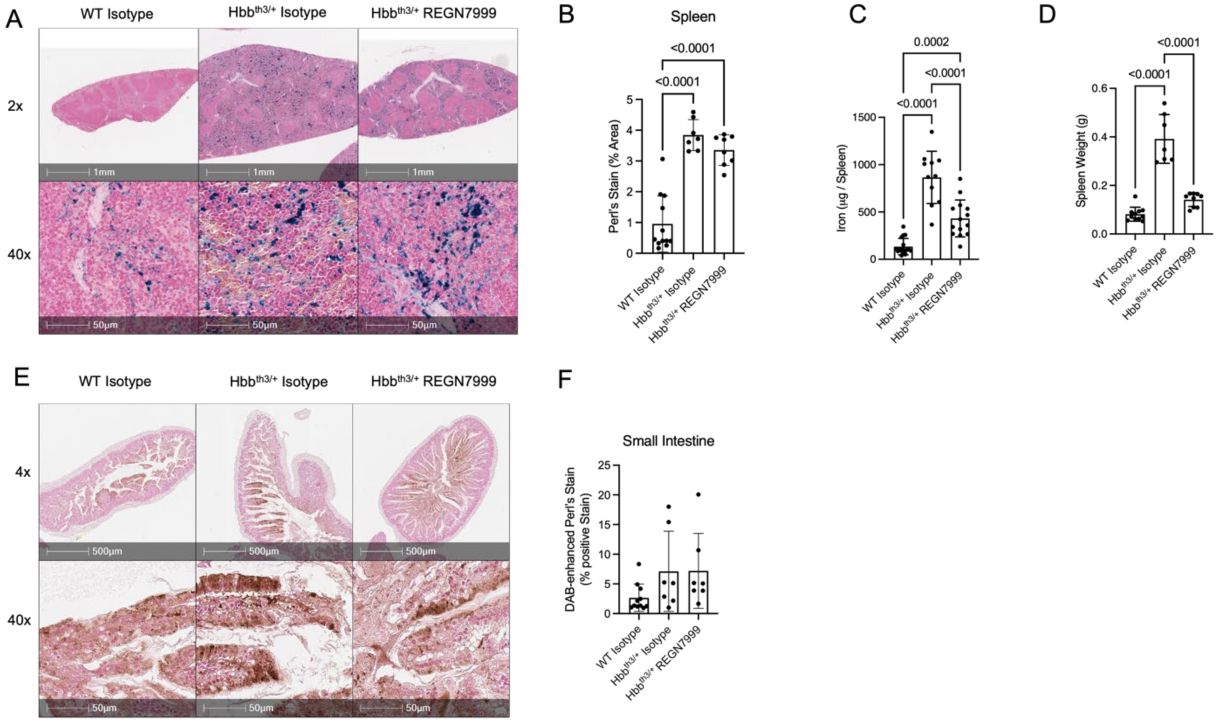

Statistics were calculated with 1-way ANOVA using Tukey's post-hoc test. A statistically significant difference was reached at $P < 0.05$.

Supplemental Figure 9. Treatment with REGN7999 improved RBC health in spleen and bone marrow. Carboxy-H2DCFDA fluorescence as an indicator of oxidative stress was measured in (A) spleen and (B) bone marrow of Hbb$^{th3/+}$ mice and WT littermates after 8 weeks treatment with REGN7999 or isotype control. Annexin V staining was used to assess RBC senescence in (C) spleen and (D) bone marrow from the same treatment groups.

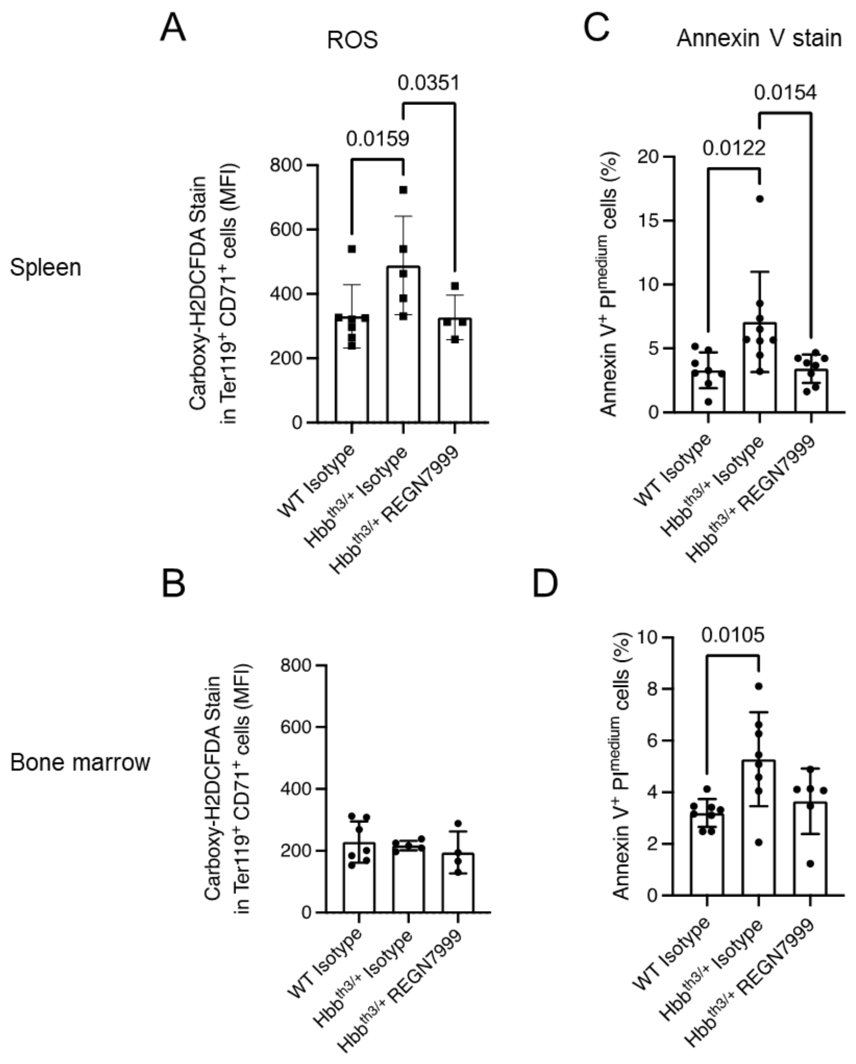

Carboxy-H2DCFDA, 6-carboxy-2’, 7’-dichlorodihydrofluorescin-diacetate; ROS, reactive oxygen species; MFI, median-fluorescent intensity.

Statistics were calculated with 1-way ANOVA using Tukey's post-hoc test. A statistically significant difference was reached at $P < 0.05$.

Supplemental Figure 10. Hbb$^{th3/+}$ mice produced more lactate and had regular lactate clearance. (A) Overview of the training plan and forced exhaustion run. (B) Running distance recorded during a forced exhaustion run from Hbb$^{th3/+}$ mice or WT littermates after REGN7999 or isotype control injections. (C) Ratio of blood lactate levels measured directly at exhaustion and running distance to receive lactate produced per meter running of the same mice. (D) Running distance of C57Bl/6 mice after 8 weeks of REGN7999 or isotype control injections. (E) Basal blood lactate of Hbb$^{th3/+}$ mice or WT littermates. (F) Blood lactate levels over 2 h in Hbb$^{th3/+}$ mice or WT littermates after receiving a bolus injection of lactate to measure lactate tolerance. (G) Hepatic and (H) erythrocyte LDH activity in Hbb$^{th3/+}$ mice or WT littermates.

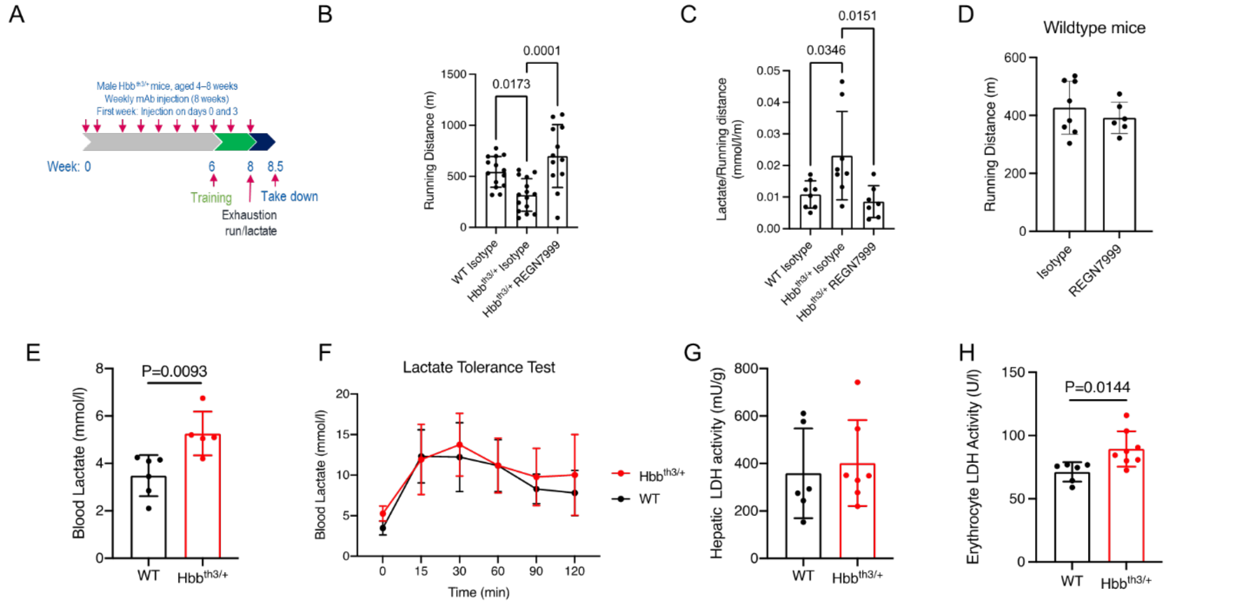

LDH, lactate dehydrogenase.

Statistics were calculated with 1-way ANOVA using Tukey's post-hoc test. A statistically significant difference was reached at $P < 0.05$.

Supplemental Figure 11. Spleen volume reduction and secondary erythropoiesis improvement preceded bone health improvement. (A) Representative pictures of spleen measurements in mice in sagittal orientation. (B) Longitudinal MRI showing spleen volume in WT, Hbb$^{th3/+}$ isotype control, and REGN7999 injected mice. (C) Bone volume and (D) bone mineral density after 4 weeks of REGN7999 injection.

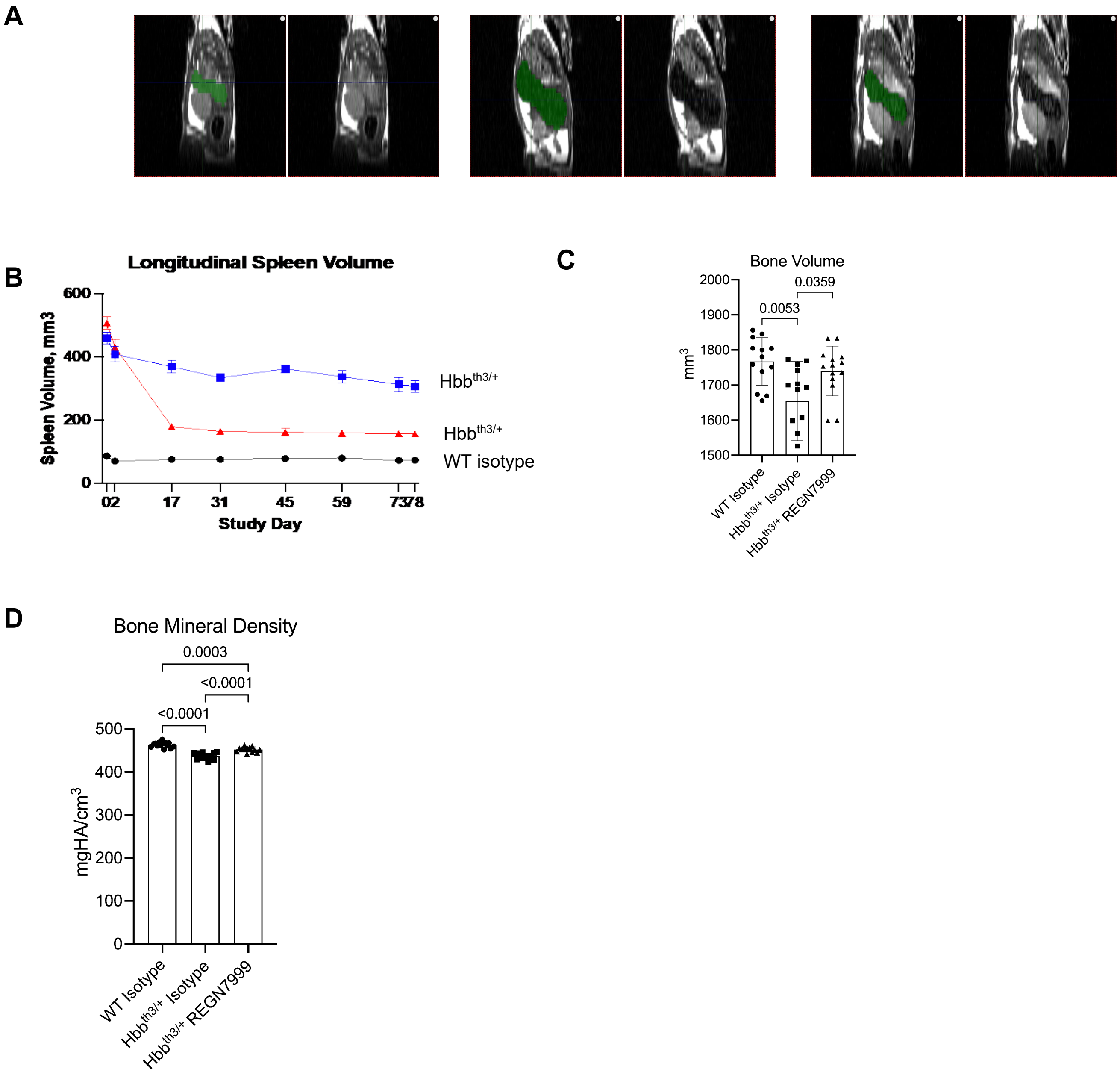

Supplemental Figure 12. REGN7999 but not Acvr2b(L79D)-Fc reduced spleen weights in Hbb$^{th3/+}$ mice after 8 weeks of treatment.

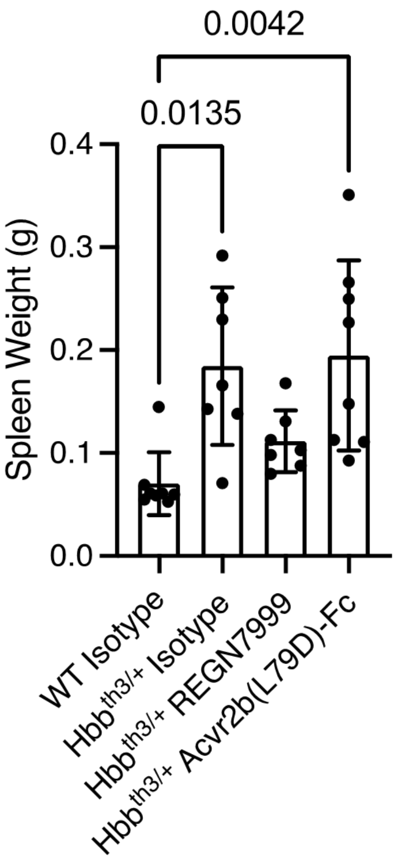

Statistics were calculated with 1-way ANOVA using Tukey's post-hoc test. A statistically significant difference was reached at $P < 0.05$.

Supplemental Figure 13. Disposition of the study participants in the single-ascending dose phase I study of REGN7999

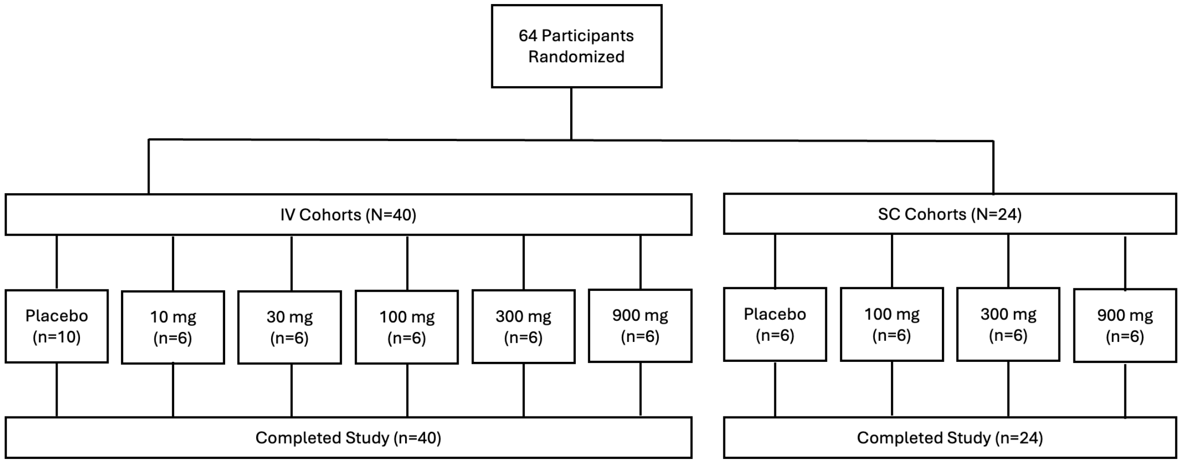

Supplemental Figure 14. REGN7999 administration reduces serum iron and increases hepcidin within 4 days in healthy clinical trial participants. In the first-in-human study, REGN7999 reduced serum iron independent of (A) i.v. or (B) s.c. injection. In contrast, serum hepcidin levels increased in a dose-dependent manner after (C) i.v. and (D) s.c. injection. Biomarkers were assessed at 4, 8, and 24 hours post-dose for i.v. and 24 hours post dose for s.c.

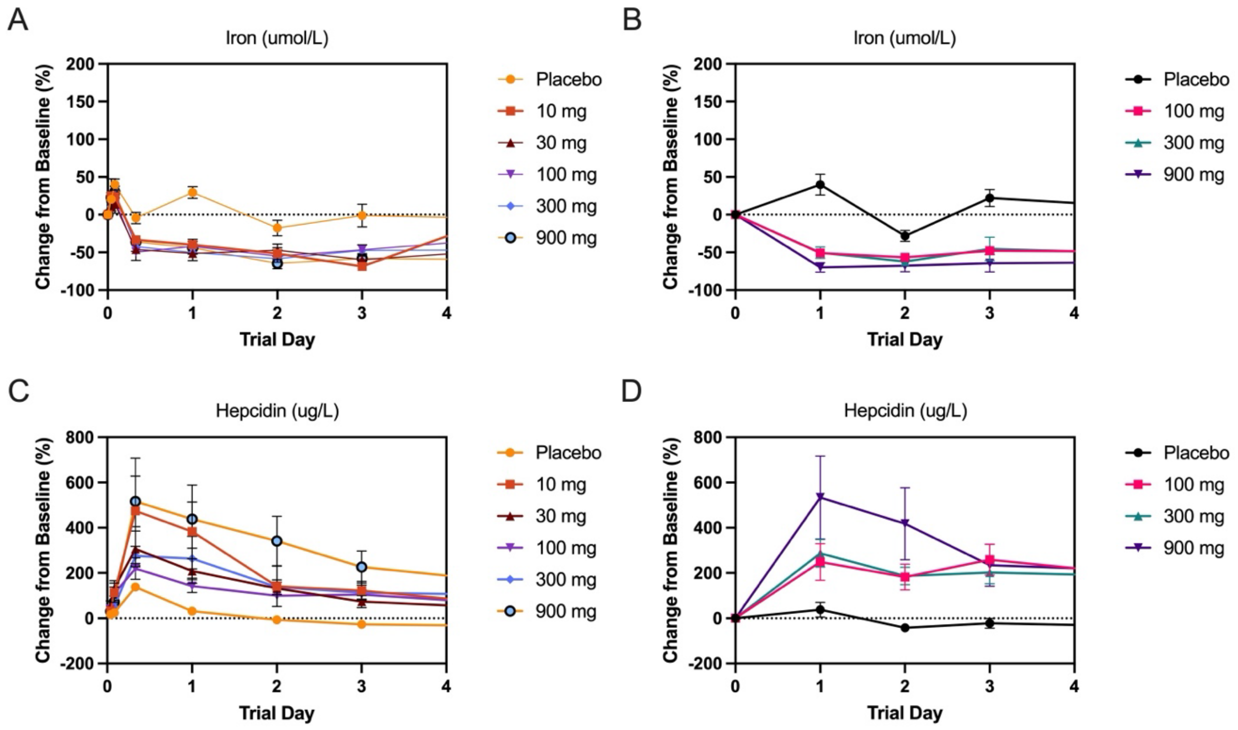

Supplemental Figure 15. REGN7999 administration in the first-in-human study reduced transferrin saturation within 4 days independent of (A) i.v. or (B) s.c. administration. In contrast, serum ferritin levels increased within 4 days in a dose-dependent manner after (C) i.v. and (D) s.c. administration. Biomarkers were assessed at 4, 8, and 24 hours post-dose for i.v. and 24 hours post-dose for s.c. cohorts.

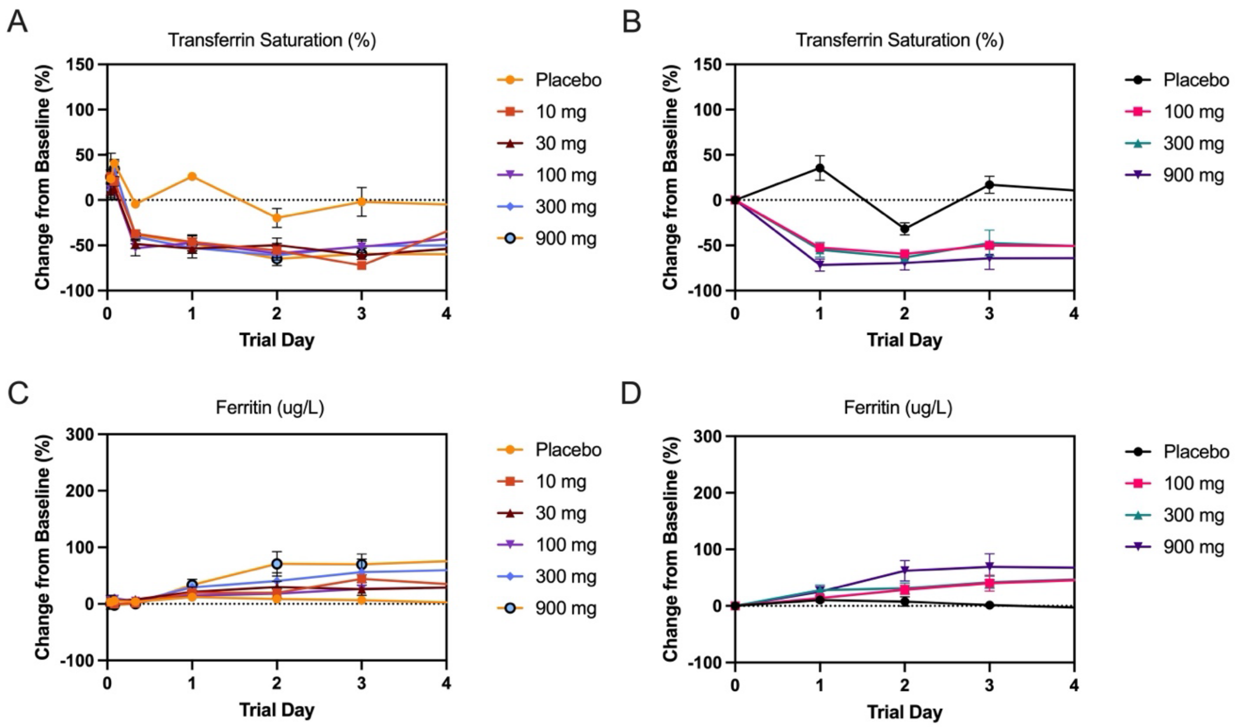

Supplemental Figure 16. Decreases in (A, B) MCV, (C, D) reticulocyte count, and (E, F) reticulocyte hemoglobin through the end of the study were consistent across i.v. and s.c. doses.

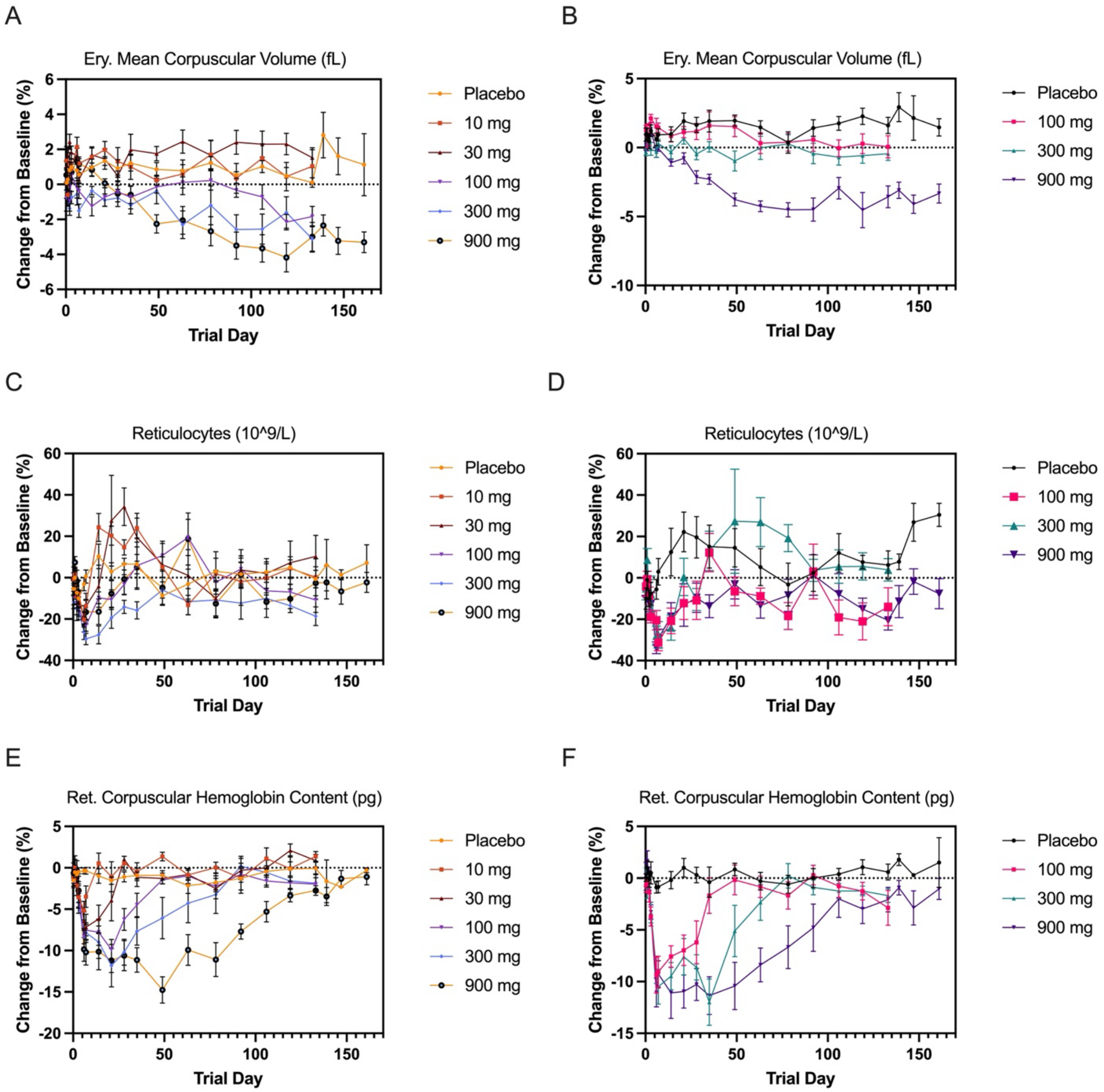

MCV, mean RBC corpuscular volume.
